# BACH1 orchestrates macrophage state transitions to coordinate regenerative inflammation

**DOI:** 10.1093/jimmun/vkag101

**Published:** 2026-06-10

**Authors:** Noemí Caballero-Sánchez, Petros Tzerpos, Krisztian Bene, Laszlo Halasz, Dóra Bojcsuk, Gergely Nagy, Maysaa A Ali, Timea Cseh, Szilard Poliska, Matthew J Borok, Jordan Scherer, Frederic Relaix, Enrique Saez, Zsolt Czimmerer, Andreas Patsalos, Laszlo Nagy

**Affiliations:** Doctoral School of Molecular Cell and Immunobiology, Faculty of Medicine, University of Debrecen, Hungary; Department of Biochemistry and Molecular Biology, Nuclear Receptor Research Laboratory, Faculty of Medicine, University of Debrecen, Hungary; Department of Biochemistry and Molecular Biology, Nuclear Receptor Research Laboratory, Faculty of Medicine, University of Debrecen, Hungary; Department of Biochemistry and Molecular Biology, Nuclear Receptor Research Laboratory, Faculty of Medicine, University of Debrecen, Hungary; Departments of Medicine and Biological Chemistry, Johns Hopkins University School of Medicine, Institute for Fundamental Biomedical Research, Johns Hopkins All Children’s Hospital, St. Petersburg, FL, United States; Departments of Medicine and Biological Chemistry, Johns Hopkins University School of Medicine, Institute for Fundamental Biomedical Research, Johns Hopkins All Children’s Hospital, St. Petersburg, FL, United States; Department of Biochemistry and Molecular Biology, Nuclear Receptor Research Laboratory, Faculty of Medicine, University of Debrecen, Hungary; Department of Biochemistry and Molecular Biology, Nuclear Receptor Research Laboratory, Faculty of Medicine, University of Debrecen, Hungary; Doctoral School of Molecular Cell and Immunobiology, Faculty of Medicine, University of Debrecen, Hungary; Department of Biochemistry and Molecular Biology, Nuclear Receptor Research Laboratory, Faculty of Medicine, University of Debrecen, Hungary; Department of Biotechnology, College of Applied Sciences, University of Technology, Baghdad, Iraq; Department of Biochemistry and Molecular Biology, Nuclear Receptor Research Laboratory, Faculty of Medicine, University of Debrecen, Hungary; Genomic Medicine and Bioinformatics Core Facility, Department of Biochemistry and Molecular Biology, Faculty of Medicine, University of Debrecen, Debrecen, Hungary; INSERM U955 IMRB, University Paris-Est Créteil, Créteil, France; Departments of Medicine and Biological Chemistry, Johns Hopkins University School of Medicine, Institute for Fundamental Biomedical Research, Johns Hopkins All Children’s Hospital, St. Petersburg, FL, United States; INSERM U955 IMRB, University Paris-Est Créteil, Créteil, France; École Nationale Vétérinaire d'Alfort, U955 IMRB, F-94700, Maisons-Alfort, France; EFS, U955 IMRB, F-94010, Créteil, France; Service d’Histologie, Assistance Publique-Hôpitaux de Paris, Hôpital Mondor, Créteil, France; Department of Molecular and Cellular Biology, Scripps Research, La Jolla, CA, United States; Institute of Genetics, HUN-REN Biological Research Centre, Szeged, Hungary; Departments of Medicine and Biological Chemistry, Johns Hopkins University School of Medicine, Institute for Fundamental Biomedical Research, Johns Hopkins All Children’s Hospital, St. Petersburg, FL, United States; Department of Biochemistry and Molecular Biology, Nuclear Receptor Research Laboratory, Faculty of Medicine, University of Debrecen, Hungary; Departments of Medicine and Biological Chemistry, Johns Hopkins University School of Medicine, Institute for Fundamental Biomedical Research, Johns Hopkins All Children’s Hospital, St. Petersburg, FL, United States

**Keywords:** BACH1, inflammation, muscle repair, regenerative inflammation, transcription factor

## Abstract

Efficient tissue regeneration requires the precise coordination of inflammatory and regenerative programs, principally mediated by monocyte-derived macrophages. However, the transcriptional wiring and epigenomic processes behind complex macrophage subtype specification and transition between the different states are not known. Here we have identified the transcriptional repressor BACH1 as a critical, cell-intrinsic regulator of monocyte-derived macrophage specification during skeletal muscle regeneration. Using a myeloid-specific BACH1 knockout mouse model, we demonstrate that BACH1 deficiency disrupts the temporal coordination of monocyte-to-macrophage differentiation, leading to aberrant macrophage subsets with concurrent opposing pro- and anti-inflammatory features. Single-cell RNA-sequencing profiling reveals that BACH1 controls a core transcriptional network, including *Nfkb1*, *Cebpb*, and interferon signaling, governing inflammatory resolution and functional macrophage specialization. Mechanistically, BACH1 loss accelerates macrophage differentiation but also affects its core cellular identity, resulting in sustained, rather than declining inflammatory programs including upregulation of *Il1b* and thus, defective tissue remodeling. These immune alterations compromise the paracrine landscape during regenerative inflammation and impair muscle stem cell differentiation. Our findings establish BACH1 as a molecular tuner or controller that integrates early innate immune signaling with regenerative output, positioning it as a central node linking transcriptional control, immune fate decisions, and tissue repair.

## Introduction

Tissue regeneration and repair is a complex, conserved and tightly regulated process that consists of overlapping phases of inflammation, cellular proliferation, extracellular matrix (ECM) remodeling, and the restoration of tissue architecture.^1^ Disruption of these highly integrated phases may result in pathological outcomes such as chronic wounds, fibrosis, or tumorigenesis.^2^ A successful regeneration response relies on the precise coordination of immune and stromal components, among which macrophages play a central role. During the early stages of regenerative inflammation, macrophages act as key orchestrators of the repair process.^3^ Traditionally viewed as binary (M1/M2) polarized populations,^4^ macrophages were thought to function primarily by clearing cellular debris in the early stages of injury, followed by promoting and resolving tissue-specific regenerative programs through the secretion of lipid mediators and growth factors in later phases.^5^^,^^6^ However, advances in technologies such as single-cell RNA sequencing (scRNA-seq) have dramatically expanded our understanding of macrophage biology highlighting their remarkable heterogeneity and specialized functions during different stages of repair.^7^^,^^8^ In skeletal muscle, scRNA-seq trajectory analyses have characterized the dynamic transitions and lineage relationships between these subsets during regeneration.^9–13^ For example, our recent work has identified distinct populations in CTX-injured muscle: patrolling monocytes, infiltrating pro-inflammatory monocytes/macrophages, transitional macrophages, pro-resolution macrophages, and growth factor-producing macrophages.^12^ Importantly, spatial transcriptomics has further demonstrated that the spatial localization of these macrophage subsets is crucial for effective regeneration, particularly in the Duchenne Muscular Dystrophy model, where growth factor-expressing and pro-resolution macrophages are enriched near regenerating fibers, suggesting functional compartmentalization within the tissue microenvironment.^12^

Macrophages are able and required to adjust their phenotype and activity in response to changes in the local microenvironment to function properly. This plasticity is tightly controlled at the transcriptional and epigenome level, with multiple transcription factors modulating gene expression programs that regulate monocyte recruitment, differentiation and macrophage polarization, metabolism, and effector functions.^6^^,^^14^^,^^15^ Among these transcriptional regulators, BTB and CNC Homology 1 (BACH1) has been shown as a critical factor orchestrating macrophage identity and function,^16^ acting in dual roles as a pioneer repressor and epigenomic bookmarker.^17^ Depletion of *Bach1* has been associated with attenuation of atherosclerosis,^18^ while elevated *Bach1* expression has been associated with impaired tissue regeneration,^19^^,^^20^ Alzheimer’s disease,^21^ and unfavorable prognoses in various cancers.^22–24^ Generally, these studies suggest that upon oxidative stress or increased heme levels, BACH1 is degraded, allowing for the induction of *Hmox1* and other protective genes. However, dysregulated or sustained *Bach1* expression can suppress these protective mechanisms, leading to increased oxidative damage and chronic inflammation, thereby hindering effective tissue repair.^25^^,^^26^ Despite the general maladaptive role in chronic inflammation and tumor progression of BACH1, recent studies suggest that this transcription factor is required for proper regenerative inflammation in skeletal muscle.^27^^,^^28^ The acute skeletal muscle injury model is a powerful tool for investigating the contribution of macrophages to subsequent steps of regenerative inflammation providing a well-defined temporal framework and reproducible injury patterns, allowing to meticulously dissect the dynamics of macrophage behavior during tissue regeneration.^9^^,^^29^ The apparent dichotomy, wherein BACH1 depletion drives maladaptive chronic inflammation yet BACH1 itself is essential for muscle repair, strongly suggests a context-dependent role. This indicates that BACH1 can contribute to pathological conditions in certain tissues while at the same time supporting essential regenerative processes in others. Collectively, these studies reinforce the fundamental role of macrophages in tissue repair and disease progression and identify BACH1 as a key factor regulating their diverse activities.

Our previous work using a systemic Bach1 knockout model suggested that BACH1 may play a role in macrophage-driven muscle repair, but the precise cellular and molecular mechanisms remained unresolved.^28^ In the present study, we overcome these limitations by employing a myeloid-specific conditional Bach1 knockout in combination with single-cell RNA sequencing. This approach allowed us to interrogate the transcriptional heterogeneity of infiltrating myeloid subsets with high resolution, distinguishing cell-intrinsic effects of BACH1 deficiency. Through this strategy, we identify defects in interferon-γ signaling, sustained pro-inflammatory responses at both RNA and protein levels, and impaired macrophage polarization and differentiation during muscle regeneration. Among these pathways, interleukin (IL)-1β emerges as a key BACH1-regulated factor whose dysregulation compromises muscle stem cell differentiation. Notably, our data reveal that BACH1 exerts its regulatory effects as early as day 2 post-injury (dpi2), preceding the subsequent phenotypic alterations observed by flow cytometry at dpi3 and dpi4. These findings establish BACH1 as an early transcriptional regulator coordinating the temporal balance of inflammatory and reparative programs in regenerative inflammation.

## Results

### BACH1 is indispensable for effective muscle regeneration via myeloid cell control

To investigate the role of BACH1 in myeloid cells during regenerative inflammation, we generated a novel conditional knockout (cKO) murine model. Using a CRISPR/Cas9-mediated genome editing strategy, we inserted loxP sites flanking exon 4 of the Bach1 gene, targeting a region that encodes the DNA-binding domain (DBD). Bach1^fl/fl^ mice were crossed with Lyz2-Cre transgenic mice, which express Cre recombinase under the control of the Lyz2 (LysM) promoter, enabling myeloid-specific deletion of *Bach1* in monocytes, macrophages, and neutrophils (Fig. S1A). Efficient insertion of the loxP and Cre sites was validated by genotyping (Fig. S1B), and successful gene deletion was confirmed by RNA-seq analysis of untreated bone marrow-derived macrophages (BMDMs), as shown by IGV plots (Fig. S1C). As a model of regenerative inflammation, we used cardiotoxin (CTX)-induced acute skeletal muscle injury, a well-established method for inducing sterile myofiber necrosis while preserving satellite cells and the extracellular matrix.^30^^,^^31^

**Figure 1:**
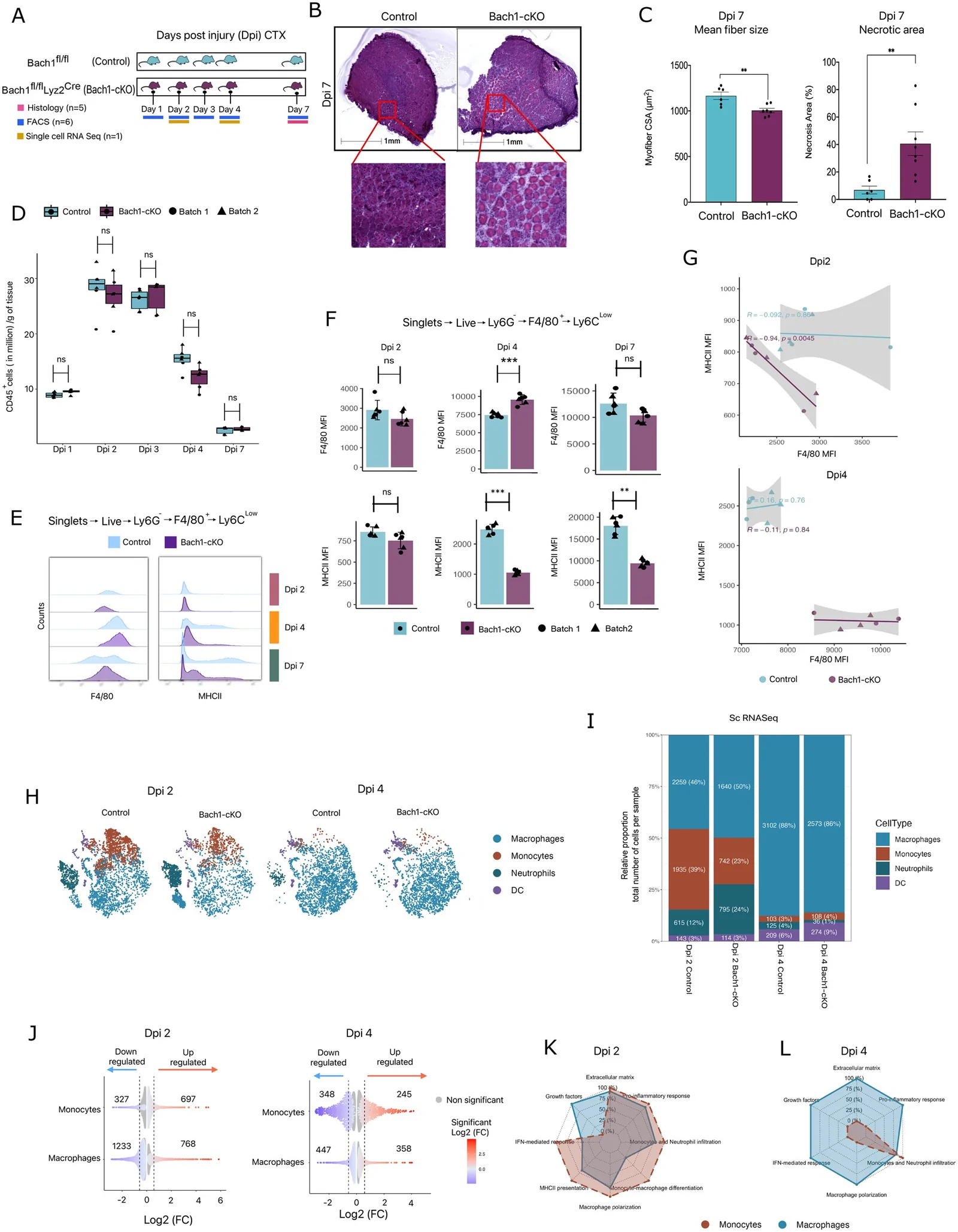
Myeloid-specific Bach1 knockout reveals impaired muscle repair though altered macrophage dynamics. (A) Schematic of the experimental design. Muscle injury was induced via cardiotoxin (CTX) injection, with analyses conducted at various days post-injury (dpi) using histology (*n* = 5/group), flow cytometry (*n* = 6/group), and single-cell RNA sequencing (*n* = 1/group). (B) Representative H&E-stained sections of regenerating tibialis anterior (TA) muscle at dpi 7 in control and Bach1-cKO mice. Scale bar: 1 mm. (C) Quantification of mean myofiber cross-sectional area (CSA) in TA muscle at dpi 7. Data represent mean ± SEM; *P* < 0.05, *P* < 0.01, *P* < 0.001 (Student unpaired *t* test). (D) Total CD45^+^ cell counts (millions per gram of tissue) in injured TA muscle at dpi 1, 2, 3, 4, and 7. Data were obtained from two independent experimental cohorts (Batch 1 and Batch 2). Values represent mean ± SEM; ns, not significant (2-tailed unpaired Student *t* test). (E) Representative histograms of F4/80 and MHCII mean fluorescence intensity (MFI) in control and Bach1-cKO mice across time points. (F) Quantification of F4/80 and MHCII MFI at dpi 2, 4, and 7 from 2 independent cohorts (Batch1 and Batch2). Data represent mean ± SEM; *P* < 0.05, *P* < 0.01, *P* < 0.001 (Student unpaired *t* test). (G) Correlation plot showing the relationship between F4/80 and MHCII MFI. (H) t-SNE plot of single-cell RNA-seq data clustered based on annotated cell type populations. (I) Relative abundance of immune cell populations expressed as a proportion of total recovered cells per sample (dpi 2 and 4; control versus Bach1-cKO). (J) Volcano plots showing differentially expressed genes (DEGs) in macrophages and monocytes at dpi 2 and 4. Red: upregulated in Bach1-cKO; blue: downregulated. (K–L) Gene Ontology (GO) enrichment analysis of DEGs between Bach1-cKO and control macrophages, shown as spider plots.

To assess the impact of *Bach1* loss in the conditional Bach1-cKO, we established an experimental timeline integrating histological, immunological, and transcriptomic analyses at defined time points post-injury in Bach1^fl/fl^ (control) and Bach1^fl/fl^Lyz2^Cre^ (Bach1-cKO) mice (Fig. 1A). At day 7 post-injury (dpi 7), hematoxylin and eosin (H&E) staining revealed a higher frequency of necrotic muscle fibers in Bach1-cKO mice, characterized by an absence of central nuclei, pale cytoplasm, and fragmentation (Fig. 1B). Quantification, normalized to tissue area of center nucleated myofibers, showed a 33.76% ± 10.32% increase in necrotic fibers in Bach1-cKO mice compared to controls (Fig. 1C). In parallel, the proportion of healthy, nucleated fibers was significantly lower in the Bach1-cKO group (Fig. 1C), suggesting a delay in muscle regeneration associated with *Bach1* loss in myeloid cells. To determine whether impaired regeneration in Bach1-cKO mice was associated with altered immune dynamics, we quantified CD45^+^ cells in the injured muscle over the course of the regenerative process (dpi 1, 2, 3, 4, and 7). The experiments were performed in two independent cohorts (Batch 1 and Batch 2), obtaining comparable results. No significant differences in the total number of infiltrating immune cells between control and Bach1-cKO mice (Fig. 1D) supporting the notion that infiltration per se is unlikely to be impacted. To further dissect potential changes in myeloid cell behavior, we performed flow cytometry on CD45^+^ cells isolated from injured skeletal muscle at the same time points (dpi 1, 2, 3, 4, and 7). Using established gating strategies,^32^ we identified monocyte-derived macrophages and characterized them based on Ly6C and F4/80 expression (Fig. S1D). While both control and Bach1-cKO mice exhibited similar levels of Ly6C^High^ inflammatory macrophages at day 1, the knockout mice showed an accelerated shift toward Ly6C^Low^F4/80^High^ macrophages over subsequent days (Fig. S1D and Fig. S1E). Focusing on the emerging Ly6C^Low^F4/80^+^ macrophage population, we analyzed the surface expression of F4/80, a marker of macrophage differentiation, and MHCII, which has been described as highly expressed in a particular M2 macrophage subset within the CTX model^33^ (Fig. 1E). Using the same 2 independent cohorts as for the CD45^+^ cell quantification, we analyzed F4/80 and MHCII expression at dpi 2, 4, and 7 (Fig. 1F). At dpi4, F4/80 expression was higher in Bach1-cKO mice compared to controls, suggesting an accelerated macrophage differentiation process in the absence of BACH1. By dpi7, this difference was no longer evident (*t* test, *P* < 0.05; ns = not significant), indicative of a transient effect (Fig. 1F). To further characterize this population, we compared MHCII expression at early (dpi2) and later (dpi4 and dpi7) stages of differentiation (Fig. 1F). At dpi2, no significant differences in MHCII expression were observed between genotypes. However, by dpi4 and dpi7, control mice showed higher MHCII expression despite lower F4/80 MFI relative to Bach1-cKO mice (*t* test, *P* < 0.01). In control mice, F4/80 and MHCII expression exhibited a positive correlation (slope = 0.16), consistent with coordinated upregulation (Fig 1G). In contrast, Bach1-cKO macrophages displayed reduced MHCII levels and a slightly negative correlation between F4/80 and MHCII expression (slope = -0.11) (Fig 1G), suggesting disrupted coregulation of these markers in the absence of BACH1.

To better understand the altered transition into the Ly6C^Low^ reparative phenotype, we performed single-cell RNA sequencing (scRNA-seq) on CD45^+^ immune cells isolated from injured skeletal muscle at dpi2 which marks the beginning of the accelerated transition, and dpi4, representing the final stage of cellular changes. Focusing on the myeloid compartment, cellular heterogeneity was visualized using t-distributed stochastic neighbor embedding (t-SNE), which revealed distinct clusters corresponding to monocytes, macrophages, neutrophils, and dendritic cells (Fig. 1H). Cell types were annotated based on canonical markers: *Klrd1* and MHCII-associated genes (eg. H2-Oa) for dendritic cells; *S100a8*, *S100a9*, and *Cxcr2* for neutrophils; *Vcan*, *Chil3*, and *Plac8* for monocytes and *Apoe*, Mertk, *Igf1*, and *Gpnmb* for macrophages (Fig. S1F). Comparative analysis between Bach1-cKO and control mice revealed marked shifts in cellular composition, both in absolute cell numbers (Fig. S1G) and relative proportions (Fig. 1I). Analysis of single-cell RNA sequencing data (Fig. 1I) revealed relative cell type proportions that were largely concordant with flow cytometry findings (Fig. S1H). Notably, these differences in cellular composition were most pronounced at dpi2 characterized by a significant reduction in monocytes and an expansion of neutrophils in Bach1-cKO mice. Focusing on the predominant cell types, monocytes and macrophages, we identified distinct transcriptional profiles at both dpi2 and dpi4 (Fig. 1J, Table S1), with additional analyses of neutrophils and dendritic cells presented in Fig. S1I–J. Pathway enrichment analysis of differentially expressed genes revealed broad dysregulation of key functions related to myeloid cell-mediated tissue repair. These included monocyte-to-macrophage differentiation, extracellular matrix remodeling, and macrophage polarization, as well as pathways associated with pro-inflammatory signaling, MHCII antigen presentation, and growth factor signaling (Fig. 1K–L). While these disruptions were evident in both monocytes and macrophages at dpi2, by dpi4 they became predominantly restricted to the macrophage compartment, suggesting a progressive divergence in functional maturation. In addition, given the use of the Bach1^fl/fl^LysM^Cre^-tdTomato reporter line, we verified the efficiency and distribution of recombination across myeloid subsets. This model encodes a floxed stop cassette upstream of the tdTomato reporter, enabling assessment of Cre-mediated recombination by fluorescence intensity. As shown in Fig. S1K, neutrophils (Ly6G^+^) as well as monocytes and macrophages (Ly6G^−^) exhibited a clear shift in tdTomato mean fluorescence intensity compared to controls, demonstrating effective recombination in all three populations. Consistently, a Venn diagram (Fig. S1L) illustrates the overlap of deregulated genes across neutrophils, monocytes, and macrophages, while Fig. S2 details the Gene Ontology (GO) terms enriched in the shared and cell type-specific gene sets. Notably, analysis of the shared deregulated gene set across neutrophils, monocytes, and/or macrophages revealed a coordinated type I interferon transcriptional program. This signature was characterized by upregulation of key upstream regulators such as *Irf7* and *Stat1*/*Stat2*, together with canonical interferon-stimulated genes (ISGs) including *Isg15*, *Ifit1/2/3*, *Mx1*, *Isg20*, *Usp18*, and multiple Gbp family members. Additional enrichment of cytosolic nucleic acid sensors and downstream mediators such as *Ifih1*, *Dhx58*, and *Ticam1* further supports activation of innate antiviral signaling pathways. Given that skeletal muscle injury represents a sterile inflammatory context, these findings suggest aberrant interferon signaling rather than bona fide antiviral responses, indicative of dysregulated danger-sensing pathways in Bach1-cKO myeloid cells. Within macrophages specifically, we observed strong enrichment of pro-inflammatory mediators including *Il1b*, *Tnf*, *Ptgs2*, *Nlrp3*, *Trem1*, *Ccl3*, and *Ccl4*, together with pattern-recognition receptors such as *Tlr3*, *Tlr6*, and *Tlr9*, indicating amplification of inflammatory signaling cascades. In parallel, macrophages displayed upregulation of metabolic and inflammatory rewiring genes such as *Irg1* (*Acod1*) and *Slc16a3*, consistent with sustained inflammatory polarization. Strikingly, macrophage-specific enrichment of mitotic and G2/M cell cycle regulators, including *Ccnb1*, *Ccna2*, *Plk1*, *Birc5*, *Cenpe*, and *Rrm2*, suggests prolonged proliferative signaling and impaired terminal differentiation, pointing to altered maturation dynamics in the absence of BACH1. In addition, deregulation of genes involved in redox and iron metabolism, including *Hmox1*, *Slc40a1*, and *Txnrd1*, aligns with the established role of BACH1 as a regulator of oxidative stress and heme-responsive pathways. Collectively, these data indicate that *Bach1* deletion in myeloid cells drives a combined aberrant interferon inflammatory state, enhanced innate immune activation, altered metabolic programming, and disrupted macrophage maturation, ultimately compromising the coordinated transition toward a reparative phenotype during muscle regeneration. Although neutrophils were affected, the dominant and functionally consequential alterations occurred within the monocyte-macrophage lineage, where BACH1 loss reshaped both pro-inflammatory activation and subsequent pro-regenerative transitions. Consequently, the present study centers on monocytes and macrophages as the principal drivers of the regenerative phenotype.

### BACH1 at day 2 post-injury regulates monocyte and macrophage fate through transcriptional and chromatin accessibility

**Figure 2:**
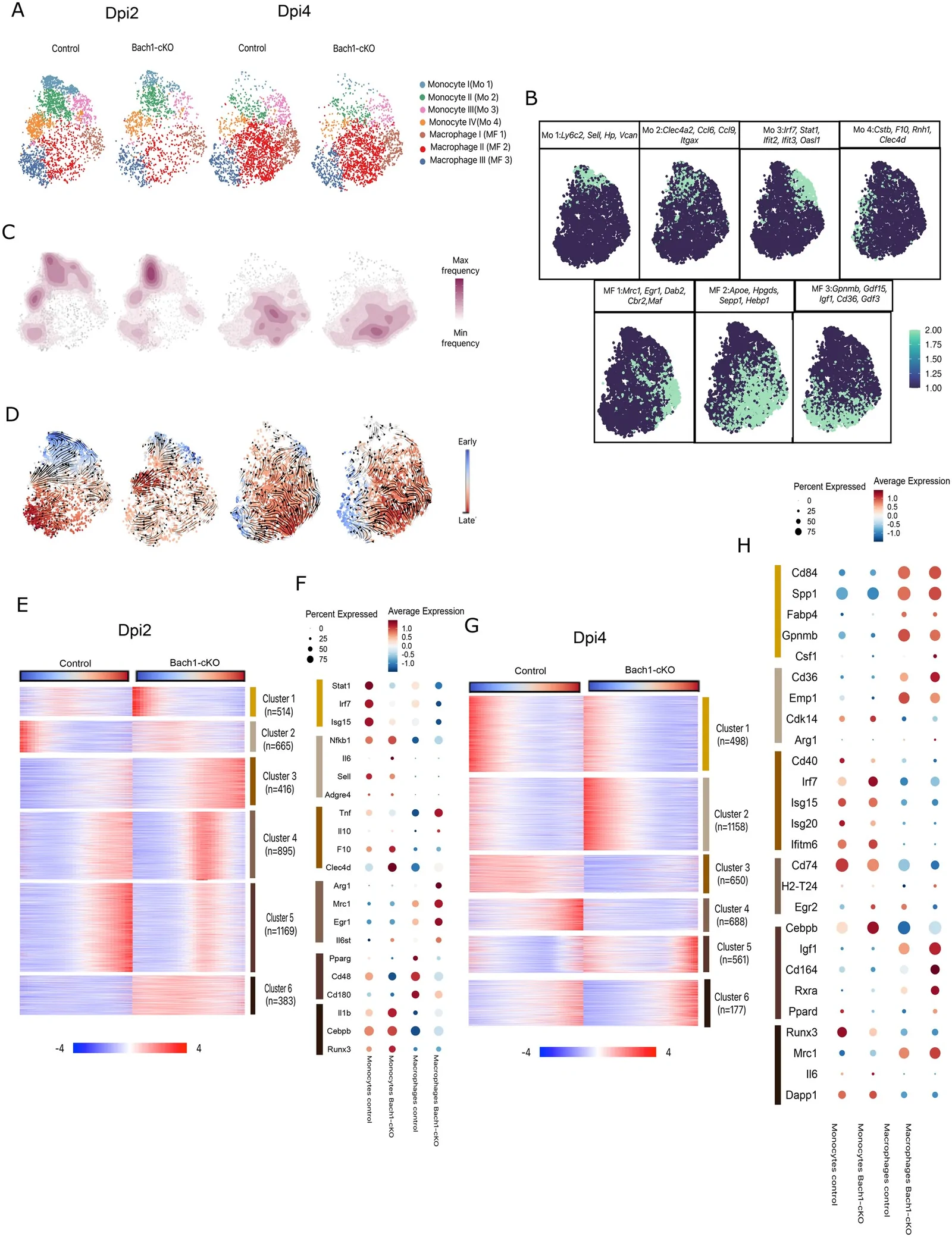
BACH1 drives early myeloid cell responses during injury. (A) Sub-clustering of myeloid cells from control and Bach1-cKO injured muscles at dpi 2 and dpi 4 visualized as t-SNE plots (B) Combined gene expression as module score for each of the identified myeloid subclusters, highlighting relevant marker genes for each cluster. (C) Density plots illustrating the cell abundance within the identified t-SNE subclusters for control and Bach1-cKO mice at dpi 2 and dpi 4. (D) Pseudotime trajectory analysis displayed as a t-SNE plot, showing the differentiation path from early (blue) to late (red) myeloid events at dpi 2 and dpi 4. (E) Heatmap of DEGs between control and Bach1-cKO samples across myeloid subclusters identified at dpi 2, ordered by pseudotime progression. (F) Dot plot of selected representative gene expression levels within identified myeloid clusters at dpi 2. (G) Heatmap of DEGs between control and Bach1-cKO samples across myeloid subclusters identified at dpi 4, ordered by pseudotime progression (H) Dot plot of selected representative gene expression levels within identified myeloid clusters at dpi 4.

To study in greater detail the alterations within the monocyte-macrophage axis, we performed subclustering of monocytes and macrophages using an optimal clustering approach combined with supervised annotation based on a curated set of literature-derived marker genes. This analysis resolved in 4 transcriptionally distinct monocyte states (Mo1–Mo4) and 3 macrophage subsets (MF1–MF3) (Fig. 2A). Each monocyte state exhibited a unique gene expression profile: Mo1 cells (*Ly6c2*^+^, *Sell*^+^, *Hp*^+^, *Vcan*^+^) corresponded to inflammatory monocytes; Mo2 (*Clec4a1*^+^, *Ccl6*^+^, *Ccl9*^+^, *Itgax*^+^) displayed signatures linked to antigen presentation and cell migration; Mo3 (*Irf7*^+^, *Stat1*^+^, *Ifit2*^+^, *Ifit3*^+^) was enriched in interferon-stimulated genes; and Mo4 (*Cstb*^+^, *F10*^+^, *Clec4d*^+^) expressed markers associated with anti-inflammatory and tissue-remodeling functions (Fig. 2B, Table S2). The macrophage compartment was similarly heterogeneous: MF1 (*Mrc1*^+^, *Dab2*^+^, *Maf*^+^) exhibited an anti-inflammatory profile; MF2 (*Apoe*^+^, *Hpgds*^+^, *Sepp1*^+^) was enriched in genes related to lipid metabolism and resolution program; and MF3 (*Gpnmb*^+^, *Gdf15*^+^, *Igf1*^+^, *Cd36*^+^) showed high expression of genes involved in growth factor production and tissue regeneration (Fig. 2B, Table S2). Although no unique clusters became apparent specifically in the Bach1-cKO condition, the relative abundance of cells across clusters are different between genotypes (Fig. 2C, Fig S3D). In Bach1-cKO mice, monocytes were predominantly concentrated within Mo1 and a portion of Mo2 clusters, while macrophages were enriched mainly within the MF2 subset. In contrast, control cells exhibited a more balanced distribution across all monocyte and macrophage states. Consistently, pseudotime trajectory analysis revealed clear alterations at dpi2 (Fig. 2D). Pseudotime refers to a computational estimate of the progression of individual cells along a dynamic biological process, such as differentiation, without requiring actual time-series data.^34^ In control samples, the differentiation trajectories progressed toward MF3, characterized by growth factor producing macrophages. In contrast, in the Bach1-cKO sample, the trajectories remained skewed toward the Mo4 state, associated with anti-inflammatory and tissue-remodeling features, suggesting an impaired transition from monocyte toward reparative macrophage phenotype (Fig. 2D). Notably, at dpi4 there are minimal changes, suggesting that Bach1-dependent regulation primarily occurs at dpi2.

To further dissect the BACH1-dependent transcriptional programs underlying the observed divergent differentiation paths, we performed unbiased k-means clustering on differentially expressed genes (DEGs) between control and Bach1-cKO cells across subclusters (Fig. 2E and 2G). Cells were ordered along pseudotime, representing early to late states and the expression level of these genes is represented by a blue-to-red color scale showing the patterns of DEGs plotted across this trajectory (Fig. 2E and 2G). This approach enables the evaluation of how gene expression programs evolve over pseudotime, providing insight into temporal gene regulation associated with macrophage differentiation in a BACH1-dependent manner. This analysis revealed six distinct temporal expression clusters at dpi2 (Fig. 2E), each representing stage-specific transcriptional programs significantly modulated upon Bach1 depletion. At dpi2, cluster 1 genes such as *Stat1*, *Irf7*, and *Isg15* gradually increased in control cells but were markedly downregulated in Bach1-cKO cells (Fig. 2F). Cluster 2 was enriched for inflammatory genes (*Nfkb1*, *Il6*) upregulated in Bach1-cKO monocytes, indicating early, aberrant activation of inflammatory pathways. Cluster 3 genes (*Tnf*, *Il10*, *Clec4d*) were also elevated in Bach1-cKO cells, suggesting continued inflammatory divergence. Cluster 4 included anti-inflammatory genes (*Mrc1*, *Arg1*) that increased over pseudotime and were upregulated in Bach1-cKO cells whereas in cluster 5 were downregulated. Cluster 6 comprised genes like *Pparg*, *Il1b*, *Cebpb*, and *Runx3*, either enriched in controls or persistently elevated in Bach1-cKO cells indicating a sustained inflammatory phenotype (Fig. 2F, Table S3). To assess whether these transcriptional alterations persisted during later stages of differentiation, we performed a similar analysis at dpi4 (Fig. 2G). Cluster 1 genes at this stage, including *Cd84*, *Csf1*, and *Gpnmb*, were significantly upregulated in Bach1-cKO macrophages. Cluster 2, enriched for *Cd36* and *Arg1*, also showed elevated expression in Bach1-cKO cells. Although interferon-responsive genes like *Irf7* and *Isg15* (cluster 3) remained differentially expressed, their distinction was less pronounced than at dpi2. Cluster 4 genes, including *Cd74* and *H2-T24*, exhibited high early expression in control cells but remained low in Bach1-cKO cells. Clusters 5 and 6 encompassed genes with increasing or sustained expression in late pseudotime, such as *Cebpb*, *Igf1*, *Ppard*, *Runx3*, *Mrc1*, and *Il6*, many of which were further enhanced in the Bach1-cKO condition (Fig. 2H, Table S4). These results indicate that BACH1 restrains early inflammatory gene expression and ensures proper differentiation timing. Its loss leads to a persistent hybrid inflammatory state that disrupts macrophage identity and function over time.

Consistent with its role in early transcriptional responses, *Bach1* expression strongly associates with Ly6c2, a marker of inflammatory monocytes, as observed in both t-SNE projections and average expression per cluster at dpi2 and dpi4 (Fig. S3A–C). To investigate whether these transcriptional dynamics are associated with changes in chromatin state, we analyzed ATAC-seq on sorted myeloid populations, Ly6C^High^F4/80^+^ and Ly6C^Low^F4/80^+^, at dpi2 and dpi4. At dpi2, Ly6C^High^F4/80^+^ cells^28^ displayed increased chromatin accessibility, with strong enrichment of MARE and TRE motifs, canonical BACH1-binding elements (Fig. S3E). This pattern was further supported by the top 1,000 highest-coverage peaks from ChIP-seq data from BMDMs,^35^ which confirmed BACH1 occupancy at regulatory loci relevant to the early myeloid function. Motif enrichment analysis revealed that genomic regions in clusters 1 and 5, associated with open chromatin in Ly6C^High^F4/80^+^ cells, were particularly enriched for BACH1-binding motifs, with MARE elements showing a higher motif enrichment score than TRE (Fig. S3F). A higher motif enrichment score indicates that the corresponding DNA sequence motif is more frequently and specifically found in accessible regulatory regions of these clusters than would be expected by chance, suggesting that BACH1 may preferentially bind and regulate genes through MARE motifs in this cellular context. BACH1′s regulatory activity was further examined by transcription factor footprinting analysis from all differentially open chromatin regions demonstrated increased BACH1 motif enrichment in dpi2 samples (Fig. S3G), highlighting temporally restricted engagement with accessible chromatin during the early inflammatory phase of muscle repair. Thus, BACH1 acts as a chromatin regulator primarily at dpi2, establishing early transcriptional programs whose downstream consequences persist across dpi4 and dpi7. In this way, BACH1 ensures that transient early chromatin remodeling at dpi2 is properly translated into balanced monocyte-macrophage differentiation, and its loss propagates continuous phenotypic divergence over time.

### BACH1 loss perturbs lineage connectivity and functional maturation during myeloid differentiation

**Figure 3:**
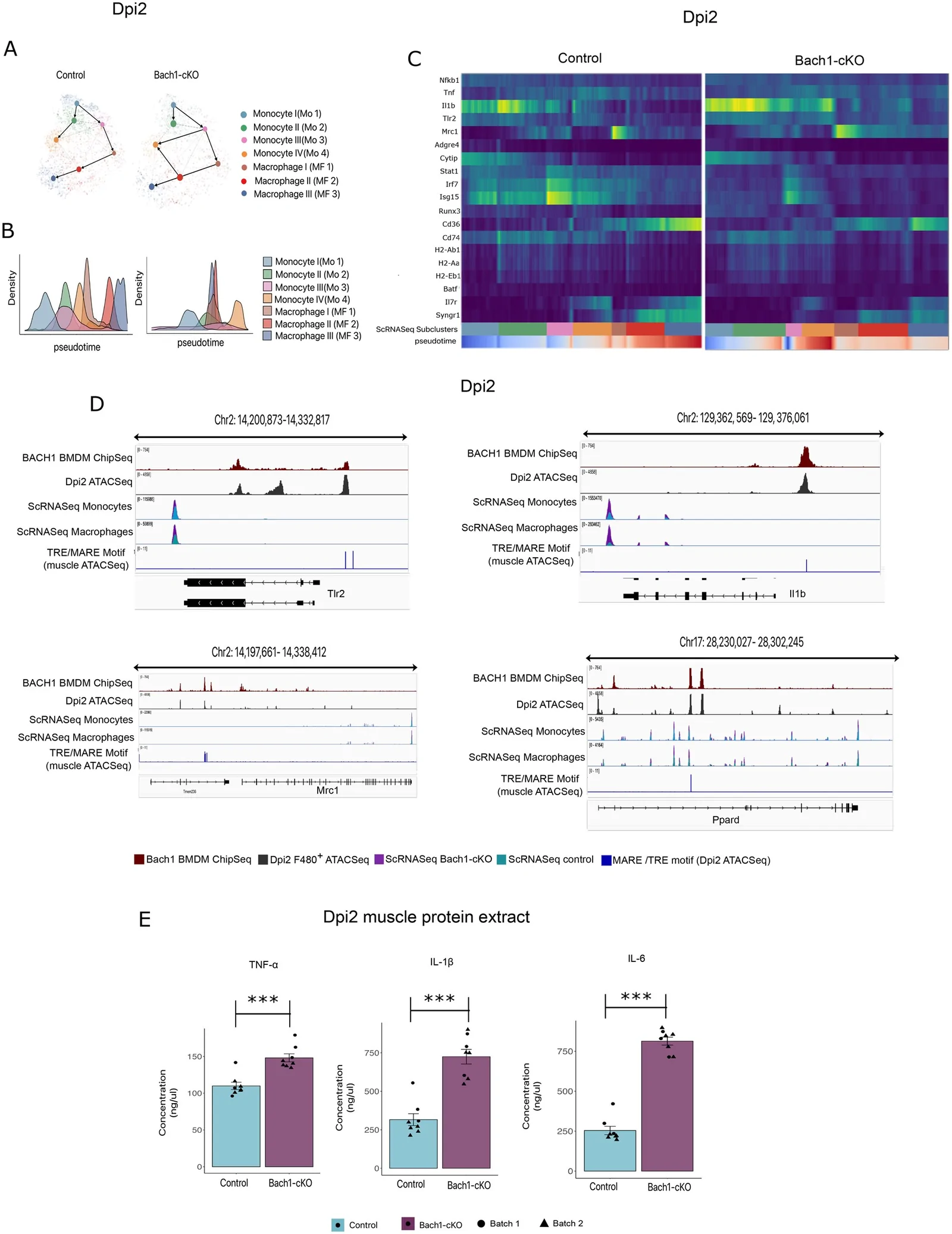
Regulatory role of BACH1 in monocyte fate disrupts macrophage differentiation and trajectory shifts. (A) Partition-based graph abstraction (PAGA) at dpi2 for control and Bach1-cKO (B) Density plots illustrating the cell abundance across pseudotime from early to late. (C) Gene expression of selected candidates across pseudotime at dpi2. Cells were ordered according to pseudotime inferred in control samples (early, blue; late, red), and Bach1-cKO cells were projected onto the same trajectory for direct comparison. (D) Genomic tracks showing Bach1 BMDM ChIP-seq data (SRR23683735), dpi 2 ATAC-seq data (GSE129393), and scRNA-seq gene expression profiles for monocytes and macrophages at specific genomic loci (eg Chr2: 14,200,873-14,332,817) (E) ELISA of TNF-α, IL-1β and IL-6 from whole muscle in control and Bach1-cKO (t-test, *P* value * < 0.05, ** < 0.01 *** < 0.001).

To mechanistically link the transcriptional reprogramming observed in Bach1-cKO monocytes and macrophages (Fig. 2) to alterations in differentiation dynamics, we applied partition-based graph abstraction (PAGA) to infer lineage relationships among transcriptionally defined myeloid states at dpi2.^36^ In control mice, the inferred trajectory showed a structured continuum originating from inflammatory monocytes (Mo1). This trajectory then diverges, leading to two distinct outcomes: one path ends in Mo4, a cluster enriched with anti-inflammatory and tissue-remodeling markers; the other, representing monocyte-to-macrophage differentiation, progresses from Mo1 to MF3, a macrophage state characterized by reparative growth factors and terminal differentiation (Fig. 3A). This transition was consistent with the pseudotemporal ordering and stage-specific gene expression programs defined previously (Fig. 2C). In contrast, Bach1-cKO resulted in a marked remodeling of the differentiation graph, characterized by disrupted connectivity and rerouted progression. Specifically, trajectories in Bach1-cKO mice failed to resolve toward MF3, instead prematurely terminating in Mo4. Notably, this divergence emerged at the MF2 bifurcation point, suggesting that BACH1 is required for the transition from lipid-resolving macrophages to reparative phenotypes. Moreover, Mo3 exhibited a bifurcated trajectory in Bach1-cKO samples, whereas in controls it followed a singular, coordinated path toward MF1, further highlighting lineage disorganization in the absence of BACH1.

Quantitative pseudotime modeling corroborated these findings, revealing an asynchronous and truncated progression of monocyte-to-macrophage differentiation in Bach1-cKO mice relative to the coordinated trajectory observed in controls (Fig. 3B). When cell density was overlaid across pseudotime, we observed that in controls, macrophage subsets emerged in a temporally ordered manner along the continuum, reflecting stage-specific differentiation programs. In contrast, Bach1-cKO macrophages collapsed prematurely into a narrow temporal window, failing to populate later stages of the trajectory. This premature convergence toward Mo4, which had been previously identified as a terminal, non-reparative macrophage state, underscores the inability of Bach1-cKO cells to engage the full differentiation spectrum. To further assess how transcriptional programs unfold along this disrupted trajectory, we projected gene expression dynamics using the pseudotime structure derived from control animals (Fig. 3C). Imposing this reference order onto the Bach1-cKO dataset revealed a striking disorganization of temporal gene regulation. In controls, inflammatory genes such as *Nfkb1*, *Tnf*, *Il1b*, and *Tlr2* were expressed early in monocytes and progressively downregulated as cells transitioned into reparative macrophages, consistent with timely resolution of inflammation. By contrast, in Bach1-cKO mice, these pro-inflammatory transcripts remained aberrantly elevated across the entire monocyte compartment, suggesting persistent activation and failure to resolve inflammatory programs. Conversely, anti-inflammatory and tissue-repair markers demonstrated an inverted pattern. The mannose receptor *Mrc1*, typically restricted to MF1 and downregulated in later macrophage stages in controls, was constitutively expressed across all macrophage states in Bach1-cKO mice, indicating a loss of temporal specificity. Similarly, *Cd36*, a scavenger receptor involved in lipid uptake and efferocytosis, displayed a sharp peak in MF3 in control animals, reflecting its potential role in late-stage resolution. In Bach1-cKO macrophages, however, *Cd36* expression remained flattened and reduced across pseudotime, failing to reach the expression levels observed in terminally differentiated reparative macrophages (Fig. 3C). Together, these data indicate that in BACH1 absence, monocyte-derived macrophages adopt a dysfunctional hybrid state, characterized by persistent inflammatory activity and impaired acquisition of reparative functions.

To determine whether the observed aberrant co-expression of pro- and anti-inflammatory programs observed in Bach1-cKO myeloid cells reflects direct regulatory control by BACH1, we integrated chromatin accessibility and transcription factor binding data with transcriptomic profiles. Specifically, we examined BACH1 occupancy (ChIP-seq in BMDMs as a proxy of bona fide BACH1 target genomic sites in myeloid cells),^37^^,^^38^ chromatin accessibility (ATAC-seq), and single-cell RNA-seq expression across monocyte and macrophage subsets. Strikingly, integrative genome viewer (IGV) snapshots revealed that several dysregulated loci harbored both BACH1 binding and accessible chromatin regions enriched in TREs and MAREs, consistent with canonical BACH1 binding motifs. For example, pro-inflammatory genes such as *Tlr2* and *Il1b* displayed BACH1 binding that coincided with increased chromatin accessibility at dpi2, and these loci were markedly upregulated in Bach1-cKO cells (purple) compared to controls (blue), across both monocyte and macrophage compartments (Fig. 3D). Similarly, anti-inflammatory and lipid metabolism-related genes, including *Mrc1* and *Ppard*, also exhibited BACH1 occupancy at regulatory regions, matched by associated ATAC-seq peaks and elevated transcription in the absence of BACH1 (Fig. 3D). These observations suggest that BACH1 directly represses both inflammatory and reparative transcriptional programs, likely safeguarding the temporal activation of these states during the different phases of myeloid polarization and differentiation. In its absence, simultaneous derepression of opposing and temporally segregated gene modules leads to a hybrid transcriptional phenotype that hampers functional inflammatory resolution and effective tissue repair.

At the functional level, this transcriptional disorganization was mirrored by the tissue cytokine milieu. Protein quantification by ELISA in whole muscle lysates at dpi2 revealed significantly elevated levels of TNF-α, IL-1β, and IL-6 in Bach1-cKO mice compared to controls, consistent with sustained activation of pro-inflammatory circuits in the absence of BACH1 (Fig. 3E). Functional validation in BMDMs further corroborated these findings (Fig. S4A). While control BMDMs responded to heme, LPS, or IFN-γ stimulation by increasing F4/80 surface expression, Bach1-cKO macrophages displayed elevated F4/80 levels even under IL-4 treatment, indicative of baseline activation and dysregulated polarization (Fig. S4B). CD86, a co-stimulatory molecule typically induced under classical activation of macrophages, is upregulated by IL-4 in Bach1-cKO BMDMs (Fig. S4C), while MHCII induction remained IFN-γ–dependent (Fig. S4D), suggesting a selective rewiring of inflammatory responsiveness. These results indicate that BACH1 is required to impose regulatory boundaries between macrophage activation states, acting as a transcriptional rheostat that tempers inflammatory responses while permitting proper engagement of reparative programs.

### Bach1 loss alters monocyte-macrophage transition independent of microenvironmental factors

**Figure 4:**
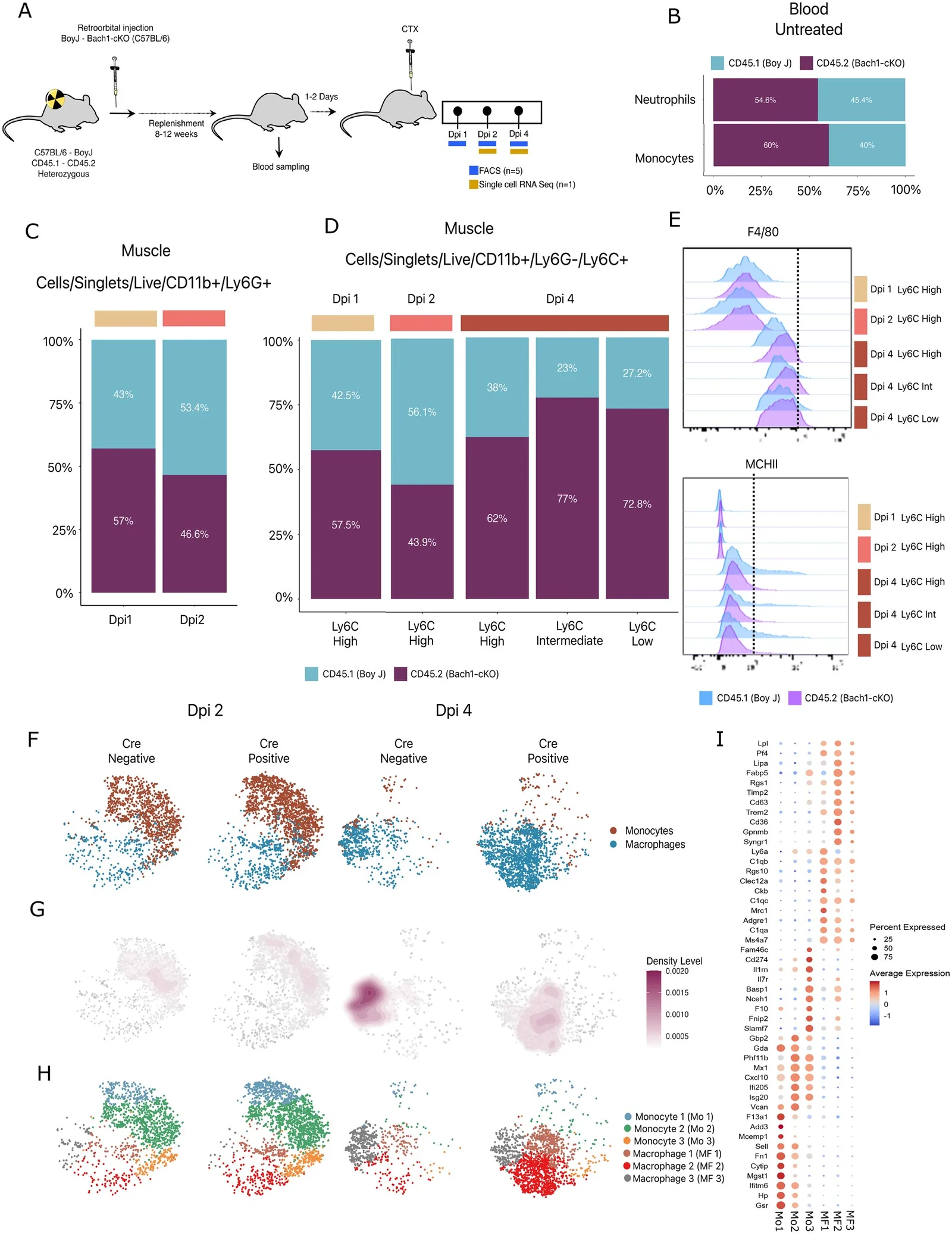
Cell-intrinsic *Bach1* deficiency alters monocyte differentiation into macrophages. (A) Schematic representation of the bone marrow chimeric model setup. (B) Representative flow cytometry plots illustrating the successful chimerism in untreated blood. Plots show the relative percentages of CD45.1^+^ (control donor-derived) and CD45.2^+^ (Bach1-cKO donor-derived) cells within the neutrophil and monocyte populations. (C) Percentage of neutrophils (CD11b^+^Ly6G^+^ cells, gated from live singlets) in the injured muscle at dpi 1 and dpi 2. (D) Flow cytometry-based quantification of the percentages of Ly6C^High^ (monocytes), Ly6C-intermediate (Ly6C^Int^), and Ly6C^Low^ (macrophages) populations within the CD11b^+^Ly6G^-^ myeloid compartment in the injured muscle at dpi 1, 2, and 4. (E) Histograms showing the expression of F4/80 and MHCII within the Ly6C^High^, Ly6C^Int^, and Ly6C^Low^ myeloid cell populations in the injured muscle. (F) t-SNE indicating cell type annotation for cre negative cells (control) and cre positive cells (Bach1-cKO). (G) Density plots illustrating the cell abundance within the identified t-SNE subclusters for cre negative cells (control) and cre positive cells (Bach1-cKO) at dpi 2 and dpi 4. (H) t-SNE plots showing the sub-clustering of myeloid cells from control and Bach1-cKO chimeric mice at dpi 2 and dpi 4. (I) Dot plot showing the percentage of abundance of cells (size) and the level of expression of each maker (blue to red scale) per subcluster.

To dissect whether the observed myeloid alterations in Bach1-cKO mice were driven by cell-intrinsic mechanisms or modulated by the inflammatory microenvironment, we generated mixed bone marrow chimeras by reconstituting lethally irradiated C57BL/6J-BoyJ recipient mice with CD45.1^+^ (WT, BoyJ) and CD45.2^+^ (Bach1-cKO) donor cells (Fig. 4A). This titrated ratio yielded approximately 60% CD45.2^+^ and 40% CD45.1^+^ monocytes in circulation post-engraftment, allowing for side-by-side comparison of WT and Bach1-cKO cells within the same inflammatory context upon CTX injury (Fig. 4B). As previously reported,^39^ whole-body irradiation effectively eliminates resident macrophages, shown by the absence of CD45.1^+^CD45.2^+^ double-positive cells (Fig. S5A) while inducing prolonged recruitment of Ly6C^High^ inflammatory monocytes (Fig. S5B compared to Fig. S1D).

**Figure 5:**
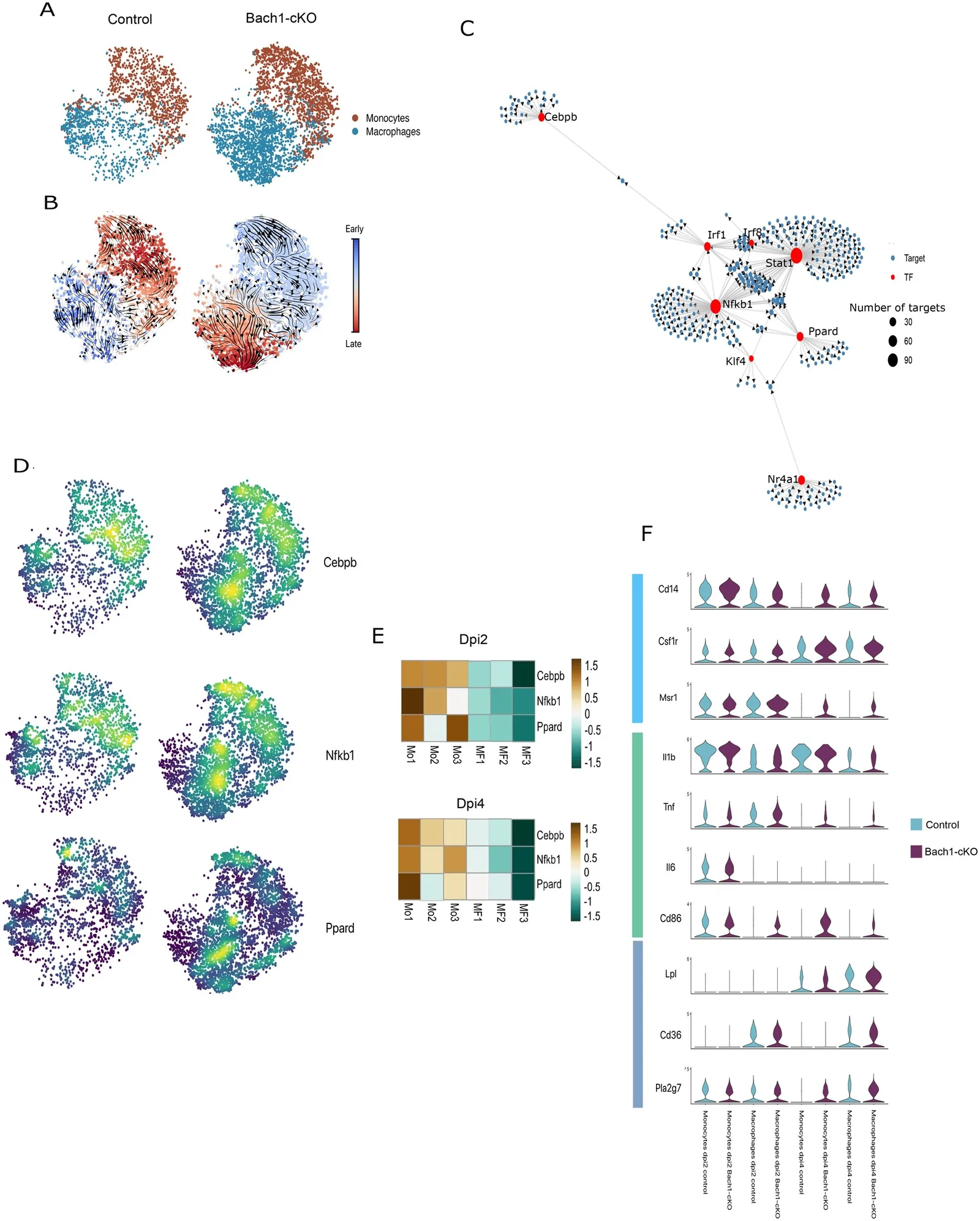
*Bach1* deficiency promotes sustained inflammatory gene expression in macrophages. (A) t-SNE of combined dpi2 and dp4 in chimeric mice for control and Bach1-cKO cells. (B) Pseudotime trajectory analysis illustrating the differentiation path of myeloid cells from early to late stages, with cells colored by their pseudotime progression, highlighting potential alterations in differentiation kinetics. (C) Network visualization representing the shortlisted transcription factor (TF)-target gene network, illustrating regulatory interactions. (D) Gene expression plots of selected transcription factors (TFs) across different myeloid cell populations (monocytes and macrophages) in chimeric mice for control and Bach1-cKO cells. (E) Pseudobulk expression of *Cebpb*, *Nfkb1* and *Ppard* across sub-clusters. (F) Violin plots showing the expression of target genes of CEBPB, NFκB and PPARD.

Initial analyses revealed no defects in leukocyte recruitment, as total CD45^+^ cell infiltration at dpi1 was comparable between genotypes even in non-chimeric mice (Fig. 1D), indicating that BACH1 deficiency does not impair cellular infiltration into injured tissue. This observation was further corroborated in the chimeric context, where the distribution of CD45.2^+^/CD45.1^+^ neutrophils (57%/43%) and monocytes (57.5%/42.5%) at dpi1 (Fig. 4D) closely mirrored the circulating monocytes proportions post-engraftment (Fig. 4B), confirming efficient infiltration of both genotypes. While the CD45.2^+^ fraction showed a mild reduction at dpi2, a notable increase was observed by dpi4, particularly during the monocyte-to-macrophage transition. Flow cytometry analysis on Ly6C expression (Fig. S5B) revealed a faster transition towards Bach1-cKO (CD45.2^+^) cells in Ly6C^Int^ and Ly6C^Low^ compartments, stages indicative of macrophage maturation. Absolute quantification using counting beads confirmed this shift, demonstrating significantly increased numbers of CD45.2^+^ cells in Ly6C^Int^ (*t* test; *P* = 0.00714) and Ly6C^Low^ (*t* test; *P* = 0.00914) fractions, with no change in Ly6C^High^ monocytes at any time-point (Fig. S5C). These changes reflect an accelerated intrinsically and in situ programmed differentiation trajectory in the absence of BACH1, consistent with the pseudotime compression observed in Fig. 3B. Further supporting a cell-intrinsic role, surface expression analyses of activation markers revealed elevated F4/80 and reduced MHCII on Bach1-cKO macrophages relative to control within the same chimeric host (Fig. 4E, Fig. S5D), phenocopying patterns observed in non-chimeric animals (Fig. 1E–F). These data support our previous observations that BACH1 acts as a critical regulator of monocyte fate and polarization, modulating both the rate of inflammatory macrophage maturation.

Next, we took advantage of our chimeric BM experimental scheme, and we sought to further characterize these phenotypic transitions with scRNA-seq. To this end, we used CTX-injured BM-chimeric mice with tdTomato-labeled Bach1-cKO and BoyJ as control and we profiled CD45^+^ cells from muscle tissue at dpi2 and dpi4. Genotype assignment was confirmed via Cre-specific transcript coverage and aligned with tdTomato and Hmox1 expression (used as marker as it is highly upregulated in BACH1 absence) (Fig. S5E), validating cell identity and annotation. Dimensionality reduction and clustering revealed a robust expansion of macrophage populations in Bach1-cKO cells (cre positive) relative to control (cre negative) (Fig. 4F–G, Fig. S5F). Notably, control macrophages predominantly localized in macrophages 3 (MF3), whereas Bach1-cKO macrophages were enriched in macrophage 1 and 2 (MF1 and 2). Further analysis of the single-cell data, as depicted in Fig. 4H, identified 3 distinct monocyte clusters (Mo1, Mo2, and Mo3) and 3 macrophage clusters (MF1, MF2, and MF4) with similar marker gene expression patterns as observed in non-chimeric mice. Specifically, Mo1 was characterized by the expression of *Sell*, *Gsr*, and *Gda* (Fig. 4I), while Mo2 showed enrichment in *Il6, Ly6c*, and *Ifi47* (Fig. 4I). Mo3 expressed markers such as *F10*, *Il7r*, and *Emp1* (Fig. 4I). MF1, populated almost exclusively by Bach1-cKO macrophages, expressed markers such as *Mrc1* and *Dab2* (Fig. 4I), consistent with tissue remodeling and alternative activation. MF2, also KO-enriched, featured elevated expression of *Arg1*, *Trem2*, *Cd36*, and *Pf4* (Fig. 4I), genes associated with metabolic adaptation and immunoregulation. In contrast, MF3, dominated by control cells, exhibited a combined transcriptional profile of MF1 and MF2 but lower expression and percentage of cells expressing those markers, some of them as important for differentiation as *Adgre1* (F4/80). These results suggest that in addition to altering the tempo of differentiation, macrophage-intrinsic BACH1 drives the shift in macrophage state transitions toward functional subsets.

### Loss of Bach1 leads to sustained macrophage-driven inflammation

To investigate the role of BACH1 in myeloid differentiation, we conducted a comparative transcriptional analysis of monocyte-to-macrophage trajectories in control and Bach1-cKO cells. Single-cell transcriptomes from both conditions were projected onto a t-distributed stochastic neighbor embedding (t-SNE) plot (Fig. 5A), revealing distinct clusters corresponding to monocytes and macrophages, confirming successful identification of these myeloid populations. RNA velocity-based pseudotime analysis (Fig. 5B) was performed on the combined dataset using a shared root and identical scaling parameters to ensure a common trajectory framework across genotypes. Cells were ordered along an inferred differentiation trajectory (blue: early, red: late), and streamlines indicated the direction of transcriptional progression. In both control (cre negative) and Bach1-cKO (cre positive) cells, pseudotime trajectories spanned the continuum from monocytes to macrophages, indicating progression along the same differentiation path. However, the distribution of pseudotime values differed between genotypes. In control cells, higher pseudotime values (red) were distributed across monocytes. In contrast, in Bach1-cKO cells, higher pseudotime values clustered with the macrophage compartment. This pattern reflects the increased abundance of macrophages at later differentiation stages in Bach1-cKO compared to control (Fig. S5F), rather than an alternative trajectory. Because RNA velocity-derived pseudotime is influenced by cell density and graph connectivity, the increased proportion of macrophages in Bach1-cKO compresses the trajectory toward terminal states, resulting in a more polarized early-to-late separation. Importantly, velocity-informed PAGA analysis (Fig. S6A) confirmed preserved directionality from monocyte clusters toward macrophages in both genotypes, while revealing a more consolidated transition into the terminal macrophage cluster in Bach1-cKO cells. Together, these analyses indicate that the absence of BACH1 does not invert the differentiation trajectory but instead promotes accelerated and more extensive monocyte-to-macrophage maturation, consistent with the observations in non-chimeric mice and supporting a role for BACH1 in restraining myeloid differentiation dynamics.

To identify genes directly regulated by BACH1 potentially explaining this phenotype and independent of external factors such as the tissue microenvironment or the prolonged pro-inflammatory response induced by irradiation,^39^ we performed a comparative analysis of differentially expressed genes (DEGs) between Bach1-cKO and control cells using pseudobulk-converted gene expression data. This analysis was conducted across two experimental settings: one using individual mice (referred to as the “non-chimeric” experiment), and the other using a chimeric bone marrow transplant (BMT) model in which both Bach1-cKO and control cells were exposed to the same muscle environment. As illustrated by principal component analysis (PCA) plots (Fig. S6B), the BMT setting revealed more robust transcriptional differences between control and Bach1-cKO groups, as well as clearer temporal resolution across dpi2 and dpi4. To identify genes consistently regulated by BACH1 deletion across experimental conditions, we focused on differentially expressed genes (DEGs) shared across clusters at dpi2 and dpi4. Venn diagram (Fig. S6C–D) revealed intersecting gene sets between the non-chimeric versus BMT and control versus Bach1-cKO comparisons: 419 DEGs in monocytes and 580 in macrophages at dpi2, and 1,033 in monocytes and 605 in macrophages at dpi4. These overlapping DEGs represent a core set of BACH1-dependent regulatory targets that are unaffected by bone marrow chimerism or tissue microenvironment, indicating cell-intrinsic transcriptional effects independent of systemic or environmental influences. These DEGs were visualized by k-means clustering in a heatmap (Fig. S6E), which revealed eight distinct expression patterns across monocytes and macrophages at dpi2 and dpi4. Notably, Cluster 2 includes both pro-inflammatory genes (eg *Nfkb1, Relb*) and anti-inflammatory genes (eg *Ppard*), and was markedly upregulated in dpi4 monocytes from Bach1-cKO mice compared to controls. Cluster 6, characterized by consistent upregulation in Bach1-cKO cells at both time points, includes classic pro-inflammatory mediators such as *Tnf* and *Cxcl14*. Gene Ontology (GO) analysis of the combined DEG sets revealed significant enrichment for pathways related to immune regulation, including “regulation of immune response” and “innate immune signaling,” in alignment with the transcriptional programs identified in non-chimeric comparisons. Furthermore, numerous transcription factors were differentially expressed, including previously highlighted BACH1 targets such as *Nfkb1*, *Rela*, and *Ppard*. Transcription factors grouped by family are shown in Fig. S6G, underscoring the broad regulatory impact of BACH1 deletion on immune gene networks.

To further dissect the regulatory networks underlying the observed accelerated differentiation and prolonged pro-inflammatory response, we carefully selected key transcription factors (TFs) for in-depth analysis. This curated set included *Cebpb* and *Nr4a1*, both critical for monocyte differentiation; *Stat1* and *Nfkb1*, which are central to both differentiation and pro-inflammatory activation in macrophages; *Irf1* and *Irf8*, involved in interferon-mediated responses; and *Pparg* and *Klf4*, known for their roles in orchestrating and maintaining anti-inflammatory states. Figure 5C illustrates the intricate network and shared targets of these TFs. The size of each TF’s node in the network directly correlates with the number of its targets that were also deregulated upon BACH1 depletion. This visual representation underscores that the impact of BACH1 absence extends beyond the TFs themselves, broadly affecting entire regulatory pathways due to the widespread deregulation of their target genes. Importantly, the immunological relevance of many of these deregulated targets has been previously validated. For instance, changes in mRNA levels of key pro-inflammatory cytokines such as *Tnf* (TNF-α), *Il1b* (IL-1β), and *Il6* (IL-6) were confirmed at the protein level by ELISA (Fig. 3E). Similarly, alterations in surface markers like CD86 were verified through surface staining (Fig. S4D), collectively reinforcing the profound impact of BACH1 deletion on both transcriptional and protein-level immune responses.

To gain further insight into the expression dynamics of these key TFs, we generated feature plots for *Cebpb*, *Nfkb1*, and *Ppard* (Fig. 5D). On these plots, a color scale from blue to yellow indicates increasing gene expression, with yellow representing the highest levels. By comparing these feature plots to the pseudotime trajectory shown in Fig. 5B, we observed distinct expression patterns for all 3 TFs between control and Bach1-cKO cells, while expression within each genotype remained consistent. In control cells, *Cebpb* and *Nfkb1* expression was predominantly restricted to the later stages of monocyte differentiation. In stark contrast, within Bach1-cKO cells, both *Cebpb* and *Nfkb1* exhibited higher expression at earlier pseudotime points compared to controls, and, critically, their expression persisted continuously into mature macrophage clusters. Interestingly, *Ppard* expression in control cells was confined to a very small subset of cells, whereas in Bach1-cKO cells, it was notably expressed across both monocytes and macrophages. Furthermore, we observed a crucial difference in co-expression: in control monocytes, *Nfkb1* and *Ppard* expression did not overlap, but remarkably, in Bach1-cKO macrophages, their expression patterns converged. This striking co-expression of TFs associated with both pro-inflammatory (*Nfkb1*) and anti-inflammatory (*Ppard*) signaling provides compelling evidence of the aberrant and dysregulated transitional state that arises upon BACH1 depletion. Interestingly, pseudobulk analysis of *Cebpb*, *Nfkb1*, and *Ppard* expression (Fig. 5D–E) reveals that these genes are most highly expressed in monocytes, further supporting the notion that BACH1 acts early during differentiation.

The dysregulation in gene expression of these transcription factors is further supported by the expression profiles of their respective target genes (Fig. 5F); downstream targets of CEBPB, including *Cd14*, *Csf1r*, and *Msr1*, were consistently upregulated in Bach1-cKO cells, with *Cd14* and *Csf1r* showing sustained upregulation throughout differentiation, and *Msr1* exhibiting macrophage-specific upregulation. Similarly, NFκB target genes such as *Tnf*, *Cd86*, and *Il1b* demonstrated increased expression, particularly in macrophages at day 2 post-injury (dpi2) and both monocytes and macrophages at dpi4. Concurrently, lipid metabolism factors regulated by PPARD, including *Lpl*, *Cd36*, and *Pla2g7*, were markedly upregulated in Bach1-cKO cells at dpi4. The imbalance we observe in Bach1-cKO macrophages, characterized by simultaneous activation of pro- and anti-inflammatory cues, disrupted IFN signaling, and abnormal TF expression, reflects a failure to transition out of an activated state, thereby compromising tissue repair outcomes.

### Bach1-deficient macrophages impair muscle stem cells differentiation during regeneration

**Figure 6:**
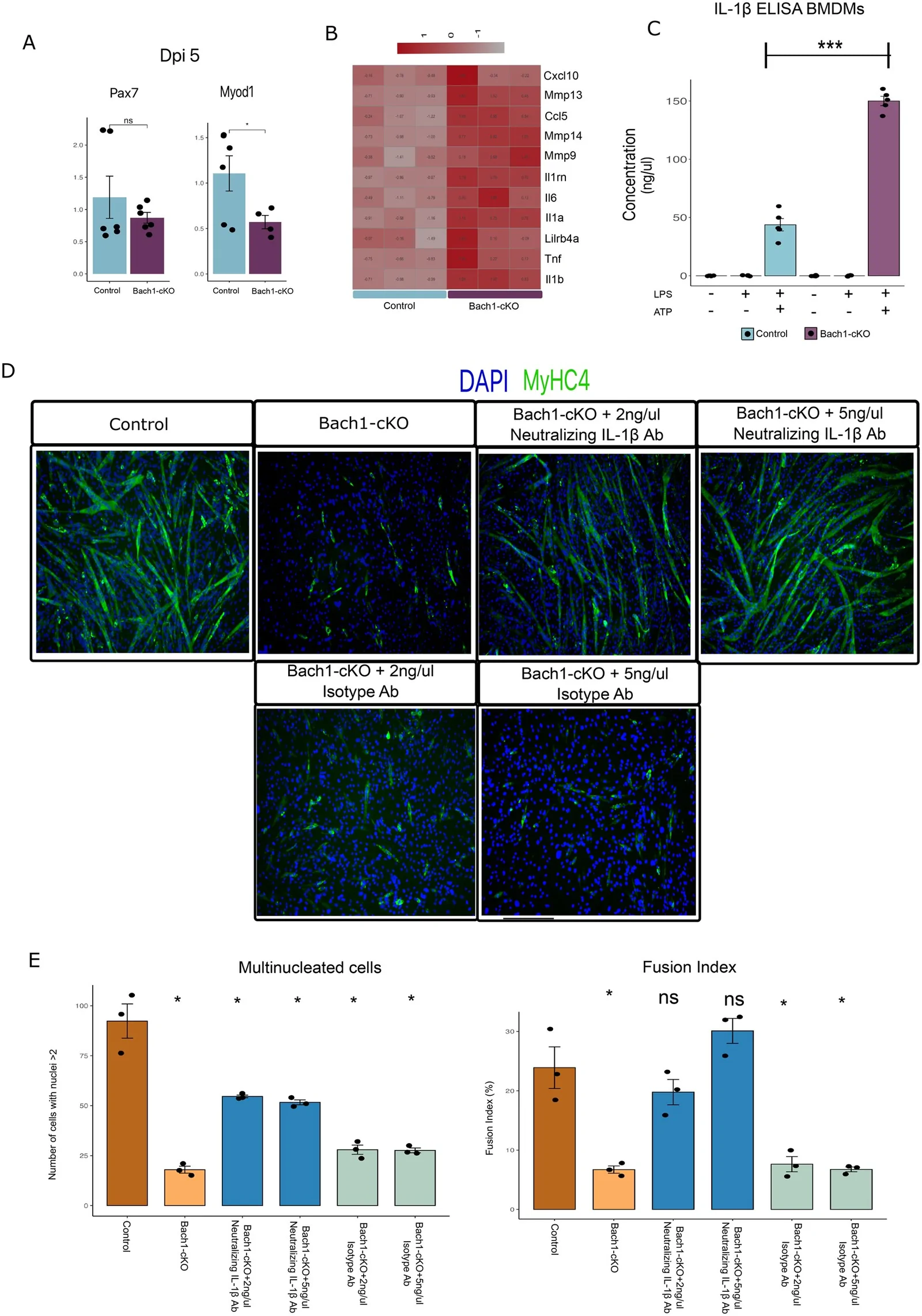
Bach1 deficiency in myeloid cells generates a pro-inflammatory microenvironment that impairs muscle stem cell differentiation. (A) qPCR analysis of Pax7 and Myod1 expression in sorted MuSCs at 5 dpi. (B) Heatmap of bulk RNA-seq data from untreated bone marrow-derived macrophages (BMDMs) of control and Bach1-cKO mice. (C) ELISA quantification of IL-1β secretion in BMDM culture supernatants under untreated conditions following LPS priming plus ATP-mediated inflammasome activation in control and Bach1-cKO cells (t-test, *P* value * < 0.05, ** < 0.01 *** < 0.001). (D) Representative fluorescence images of C2C12 myoblasts cultured in conditioned media derived from LPS-primed + ATP-activated control or Bach1-cKO BMDMs, in the presence of either neutralizing IL-1β antibody or isotype control antibody (2 ng/µl or 5 ng/µl). (E) Quantification of number of multinucleated cells and nuclear fusion index in C2C12 myoblasts from panel D (t-test, *P* value * < 0.05, ** < 0.01 *** < 0.001; control as reference).

Given the sustained pro-inflammatory state observed in the Bach1-cKO model, we investigated how this immune imbalance reshaped the muscle microenvironment and impacted muscle stem cells (MuSC) behavior. Chronic inflammation is increasingly recognized as a key regulator of stem cell function, particularly in regenerative contexts, with elevated levels of pro-inflammatory cytokines including IL-1β, known to influence stemness maintenance and modulate MuSC activation thresholds.^40–42^ In this vein, we hypothesized that the loss of BACH1 in immune cells might lead to altered paracrine signaling that influences MuSC fate decisions during regeneration. To test this, we employed NicheNet, a ligand-target inference framework, to model cell-cell communication between immune subsets^43^ and MuSCs by integrating single-cell transcriptomic publicly available data.^44^ We first identified ligands that were differentially expressed in Bach1-cKO monocytes and macrophages at dpi2 and dpi4 (Fig. S7 A and B). The potential of these ligands, including *Cxcl14*, *Il6*, or *Il1b*, to bind to MuSC receptors was subsequently classified based on inference probabilities (Fig. S7A). Notably, a fold change between control and Bach1-cKO of these ligands revealed broad upregulation in Bach1-cKO cells at dpi2, a time point critical for BACH1's observed effects (Fig. S7B). Many of these identified ligands are known mediators of inflammatory signaling, extracellular matrix remodeling, and immune cell-stem cell crosstalk, collectively suggesting a profound functional shift in the paracrine landscape of the injured muscle in Bach1-cKO mice. NicheNet predicted that these ligands would target a range of genes involved in cell cycle progression, stress response, and early activation pathways in MuSCs, including Ccnd1, *Cdk4*, *Cdkn1a*, *Fos*, and *Il6* (Fig. S7C). This pattern suggests that the immune-derived cues in the Bach1-cKO context may prematurely drive MuSCs out of quiescence and into an activated or proliferative state. Supporting this interpretation, we observed a significant downregulation of Myod1 in MuSCs sorted at dpi5 (Fig. 6A). Although there is no significant differences in the stemness of MuSCs (Fig. S7F), myod1 is a key transcriptional regulator of commitment to the myogenic lineage, and its reduced expression may reflect a disturbance in the normal activation-to-differentiation trajectory of MuSCs.

To investigate the impact of BACH1 on the macrophage secretome, we analyzed the top ligand candidates identified in Fig. S7A and performed bulk RNA-seq on untreated BMDMs. Notably, *Il1b* and *Tnf* transcripts were significantly upregulated in Bach1-cKO macrophages compared to controls (Fig. 6B), mirroring the inflammatory gene expression pattern observed in injured muscle. Importantly, this increase occurred in the absence of exogenous stimulation, indicating that BACH1 deficiency intrinsically primes macrophages toward a pro-inflammatory transcriptional state. Consistent with a direct regulatory role, our ChIP-seq analysis in BMDMs identified a BACH1 binding site proximal to the *Il1b* locus (Fig. 3), supporting the notion that BACH1 acts as a transcriptional repressor of *Il1b* under basal conditions. However, increased *Il1b* transcription does not necessarily translate into cytokine secretion, as IL-1β requires inflammasome-mediated cleavage for release. To assess functional cytokine production, we quantified IL-1β by ELISA following canonical inflammasome activation (LPS priming for 24 h followed by ATP stimulation for 15 min). Notably, upon inflammasome activation, Bach1-cKO macrophages secreted significantly higher levels of IL-1β compared to controls (Fig. 6C), demonstrating enhanced cytokine production upon inflammatory challenge.

To determine the functional consequences of this altered secretome, conditioned media (CM) from LPS primed +ATP activated BMDMs was collected. C2C12 myoblasts differentiated in CM derived from stimulated Bach1-cKO macrophages exhibited a significantly reduced nuclear fusion index compared to those cultured in control CM. Importantly, neutralization of IL-1β in the conditioned media restored myogenic differentiation (Fig. 6D and E) while isotype controls showed no difference, establishing IL-1β as a key mediator of the impaired differentiation observed under M1-polarized Bach1-deficient conditions. While these data identify IL-1β as a major effector, we acknowledge that additional inflammatory mediators may contribute to the phenotype. Accordingly, differentiation experiments using CM from untreated macrophages are presented in Fig. S7G, where the effect is detectable but less pronounced, supporting a broader inflammatory influence of BACH1 loss on the macrophage secretome.

## Discussion

Our lab in Patsalos et al.^28^ originally implicated BACH1 in muscle regeneration, demonstrating that its loss disrupted normal repair. However, the underlying mechanisms and pathways responsible for this defective regeneration and whether these effects were cell-autonomous remained unresolved. In the present study, we address this gap by employing a myeloid-specific conditional knockout in combination with single-cell RNA sequencing. This approach enabled high-resolution mapping of macrophage heterogeneity, allowing us to define discrete subsets, trace differentiation trajectories, and uncover their transcriptional programs during regenerative inflammation. Importantly, this strategy also established that BACH1’s regulatory functions are cell-intrinsic to myeloid populations rather than secondary to systemic influences.

Using high-resolution single-cell RNA sequencing (scRNA-seq), we demonstrate that BACH1 deficiency broadly impacts myeloid cell states, including neutrophils, monocytes, and macrophages. However, the most functionally consequential alterations occur within the monocyte-macrophage lineage. In this clusters, BACH1 deficiency perturbs the temporal coordination of monocyte-to-macrophage differentiation, leading to the emergence of aberrant macrophage subsets characterized by a sustained proinflammatory program. Importantly, BACH1 does not act as a global suppressor of inflammation but rather as a temporally defined and context-specific modulator of immune gene expression. In BACH1′s absence, macrophage differentiation toward Ly6C^Low^F4/80^High^ is accelerated but also aberrantly programmed, resulting in a hybrid activation state that disrupts tissue homeostasis and impairs regeneration. Flow cytometry analyses revealed altered dynamics of F4/80 and MHCII expression, consistent with disrupted co-regulation of inflammatory and resolution programs. Mechanistically, our ATAC-seq data establish that the majority of BACH1-dependent changes in chromatin accessibility, particularly at loci enriched for TRE and MARE motifs, occur as early as 2 d post-injury. This temporally precedes the phenotypic expansion of proinflammatory macrophages detected by FACS at dpi4, suggesting that BACH1 imposes regulatory control on the differentiation trajectory at an earlier stage than previously appreciated. Notably, the TRE/MARE motif enrichment closely aligns with BACH1 ChIP-seq occupancy in bone marrow-derived macrophages (BMDMs), showing direct engagement of BACH1 with these cis-regulatory elements. Here, we extend this concept by demonstrating that BACH1 governs this process in a temporally defined manner. Our data reveal that BACH1 occupies a nodal position in this process, directly modulating gene expression and chromatin accessibility at loci associated with both pro- and anti-inflammatory programs. Notably, BACH1 binds regulatory regions enriched in MARE and TRE motifs, with peak accessibility observed in Ly6C^High^F4/80^+^ cells early post-injury. This motif-specific chromatin engagement coincides with transcriptional repression of genes including *Tnf*, *Il1b*, *Mrc1* and *Ppard*, whose derepression in Bach1-cKO macrophages leads to aberrant co-expression of opposing inflammatory signatures. We show that the loss of BACH1 leads to a hyper-inflammatory state, identifying a direct link to the INF pathway affected, a central finding previously unappreciated.

Contrary to prior models suggesting that BACH1 uniformly promotes pathological inflammation, our findings support a context-dependent role. While sustained BACH1 activity has been linked to chronic inflammatory conditions such as atherosclerosis and neurodegeneration,^18^^,^^45^ here we demonstrate that transient BACH1 function is necessary for resolving acute inflammation and enabling reparative macrophage identity. This duality is echoed in recent studies of tissue-specific immune plasticity, underscoring that transcriptional regulators often play divergent roles depending on cellular context and microenvironmental cues.^46^^,^^47^ A critical question in the interpretation of these findings is whether the phenotypic changes in Bach1-cKO myeloid cells a consequence of altered tissue microenvironments are, or whether they reflect intrinsic transcriptional dysregulation. Through comparative analysis across experimental conditions, including in vitro systems and alternative injury contexts, we found that the effects of BACH1 deletion persisted independent of local cues. This observation argues in favor of a cell-autonomous mechanism, whereby BACH1 pre-configures the inflammatory potential and differentiation trajectory of monocytes prior to their engagement with tissue-derived signals. The downstream consequence of this altered inflammatory state was impaired muscle stem cell (MuSC) differentiation. Inflammatory macrophages are known to produce a suite of cytokines and growth factors that influence MuSC fate decisions, ranging from activation and proliferation to terminal differentiation. Our data show that in the absence of BACH1, the prolonged presence of pro-inflammatory macrophages correlated with reduced expression of myogenic markers in MuSCs and a failure of effective myotube formation. Consistent with these predictions, MuSCs from Bach1-cKO mice exhibited reduced *Myod1* expression, and myoblasts cultured in conditioned media from Bach1-cKO macrophages showed impaired fusion, mimicking the effects of direct IL-1β exposure. In untreated BMDMs, Bach1 deficiency was associated with elevated *Il1b* transcript levels, supporting a potential role for BACH1 as a transcriptional repressor of this gene. Upon canonical inflammasome activation with LPS and ATP, secretion of mature IL-1β protein was significantly increased in conditioned media derived from Bach1-cKO macrophages compared to controls. Notably, neutralization of IL-1β in conditioned media from inflammasome-activated Bach1-cKO macrophages restored the impaired myogenic differentiation phenotype to levels comparable to control conditioned media, demonstrating that IL-1β is a major driver of the observed effect under inflammatory conditions. However, conditioned media from untreated Bach1-cKO BMDMs already induced a measurable impairment in C2C12 differentiation, indicating that IL-1β is not the only mediator of this phenotype. Together, these findings suggest that while IL-1β is a key effector in the context of M1 polarization, additional inflammatory factors likely contribute to the broader impact of BACH1 loss on the macrophage secretome. Overall, these findings highlight how disrupted immune transcriptional states can non-cell-autonomously alter regenerative programs, a theme increasingly recognized in aging and chronic injury models.^12–14^

Collectively, our study positions BACH1 as a critical integrator of innate immune signaling and regenerative output, enforcing transcriptional order during monocyte-to-macrophage differentiation to ensure successful tissue repair. The dysregulation observed in its absence not only provides mechanistic insight into immune dysfunction during regeneration but also identifies BACH1 as a potential therapeutic target. Selective modulation of BACH1 activity may offer a means to rebalance macrophage function in disease contexts where either chronic inflammation or failed regeneration predominates. Future studies should explore the upstream regulators of BACH1 activity during injury and whether its modulation can be temporally tuned to enhance regenerative outcomes across diverse tissues.

## Materials and methods

### Mice

Male and female C57BL/6 mice, including Bach1^fl/fl^ Lyz2^Cre^ conditional knockouts, were housed under SPF conditions. Bach1^fl/fl^ mice, generated in the institute of Experimental Medicine, Budapest were crossed with Lyz2Cre mice (Dr. Attila Mocsai, Semmelweis University) and backcrossed to C57BL/6J for eight generations. Bach1^fl/fl^ littermates served as controls. All animal procedures adhered to University of Debrecen ethical guidelines (9/2023/DEMAB) using 8–12 wk-old male healthy mice. For assessment of Cre recombination efficiency, Bach1^fl/fl^ Lyz2^Cre^ mice were further crossed to a Rosa26^LSL-tdTomato^ reporter line. In this reporter strain, a loxP-flanked STOP cassette precedes the tdTomato coding sequence, enabling tdTomato expression upon Cre-mediated recombination. All experimental comparisons were performed using age-matched littermate controls.

### Muscle injury model—cardiotoxin

Cardiotoxin (12 × 10^−6 ^mol/l) from Naja pallida was injected (50 μl) into each tibialis anterior muscle of isoflurane-anesthetized mice.

### Muscle histology

Muscle samples were snap-frozen in isopentane (−160 °C). Eight-micrometer cryosections were H&E stained. A minimum of five slides per condition, each with ≥70% regenerative area, were selected. Myofiber count and measurement was performed using HALO software (Indica Labs). Regenerative fibers (center nucleated fibers) and necrotic myofibers characterized by an absence of central nuclei, pale cytoplasm, and fragmentation were analyzed using Panoramic Viewer.

### Skeletal muscle single-cell suspensions

Mice were euthanized by cervical dislocation. Tibialis anterior muscles were excised as described in prior studies.^1^^,^^2^ The excised muscles were rinsed immediately in phosphate-buffered saline (PBS; Sigma-Aldrich), minced and digested in a solution of Dulbecco’s Modified Eagle Medium (DMEM) supplemented with 2% fetal bovine serum (FBS), collagenase II (2 mg/ml; Thermo Fisher Scientific), and DNase I (100 μg/ml; Sigma-Aldrich) at 37 °C with agitation at 900 rpm for 30 min. Cell suspensions were filtered (100 µm, 40 µm) and immune cells enriched using CD45 magnetic beads (Miltenyi Biotec) according to the manufacturer’s instructions.

### Cell sorting and flow cytometry

Fc receptors were blocked using FcBlocker (Miltenyi Biotec). Cells were suspended in 1× PBS supplemented with 2% FBS. Cell viability was determined with Fixable Viability Dye eFluor 506 (Invitrogen) and surface staining was performed using the following murine-specific antibodies: Ly6C (eBioscience; HK1.4), F4/80 (eBioscience; BM8), Ly6G (BioLegend; 1A8), MHCII (BioLegend; M5/114.15.2). Cell counting was conducted with CountBright (Thermo Fisher Scientific) according to the manufacturer’s protocol. Cells were measured and sorted using a FACSAria III (BD Biosciences), and data were analyzed with Diva software (BD Biosciences) and FlowJo software v10 (Tree Star Inc.) For quantitative MFI analyses, background fluorescence was corrected by subtracting the MFI of the unstained control from the MFI of each sample.

### Adoptive cell transfers

Recipient mice were 7-wk-old CD45.1/CD45.2 congenic hybrids (C57BL/6J × BoyJ) and were lethally irradiated (11 Gy, 6 MV photons, Elekta Versa HD) to ablate bone marrow (BM). Donor BM cells from BoyJ (CD45.1) and Bach1-cKO (CD45.2) mice were isolated from femur, tibia, and humerus in RPMI, mixed 1:3 (BoyJ: Bach1-cKO), and stained for APC-anti-F4/80 (BioLegend; BM8), PE-Cy7-anti-CD11b (BioLegend; M1/70),FITC- anti-Ly6C (BioLegend; HK1.4), PerCP-Cy5.5-anti-Ly6G (BioLegend; 1A8), BV421-anti-CD45.2 (BioLegend; 104), BV711-anti-MHCII (BioLegend; M5/114.15.2), AF700-anti-CD45.1 (BioLegend; A20).Cell viability was assessed using Fixable Viability Dye eFluor 506 (Invitrogen). Results indicate a successful reconstitution with equal contributions from both donor groups. After establishing the blood ratio, we performed the acute skeletal muscle injury with the cardiotoxin model as previously described.

### Single cell RNA sequencing

Single-cell libraries were generated using the Chromium Controller and Chromium Single Cell 3′ Library & Gel Bead Kit v3.1 (10× Genomics) according to the manufacturer’s guidelines, targeting a recovery of 10,000 cells. Briefly, a cellular suspension was mixed with a master mix containing RT Reagent B, Template Switch Oligo, Reducing Agent B, and RT Enzyme C. A total of 70 µl of the master mix with cells was loaded into row 1 of the Chromium chip, while rows 2 and 3 were filled with 50 µl of gel beads and 45 µl of partitioning oil, respectively. GEM generation was completed on the Chromium Controller, and 100 µl of the resulting GEM mixture underwent reverse transcription in a C1000 Touch Thermal Cycler (Bio-Rad) GEM cleanup was performed using Dynabeads MyOne Silane beads (Thermo Fisher Scientific). Complementary DNA (cDNA) was amplified and purified using a SPRIselect Reagent Kit (Beckman Coulter), and quality was assessed using 2,100 BioAnalyzer (Agilent Technologies). Sequencing libraries were prepared following the manufacturer’s protocol and sequenced to a target depth of 20,000 read pairs per cell using paired-end sequencing on NextSeq 500 (Illumina).

### Single cell RNA analysis

Single-cell RNA-seq data were processed using Cell Ranger (10× Genomics) for demultiplexing, alignment to mm10, and UMI quantification (average 54,000 reads/cell, 50% saturation). Quality control, including filtering high mitochondrial cells (Seurat v5.0.5, miQC v1.10.0) and doublet removal (scDblFinder v1.8.0), was performed. Normalized RNA expression (Seurat’s LogNormalize) was integrated across samples with Harmony (v1.2.1). PCA on highly variable genes enabled unsupervised Leiden clustering and tSNE visualization. Cell types were annotated via SingleR (v1.10.0) with ImmGen. Differential gene expression was identified using the Wilcoxon rank-sum test with Bonferroni correction (*P* < 0.05, fold change >1.5).

For dynamic analysis, spliced/unspliced counts were estimated (Velocyto v0.6), serving as input for RNA velocity and pseudotime trajectory analysis (scVelo v0.2.2, stochastic model). PAGA was used for inter-cluster relationships. Temporal gene expression was modeled with tradeSeq (v1.16.0) using a generalized additive model (fitGAM), identifying DEGs along pseudotime via the Wald test on the top 1,500 highly variable genes. DEGs were clustered by temporal profile using factoextra (v1.0.7). NicheNet analysis was performed to predict pro-inflammatory ligands differentially expressed in Bach1-cKO monocytes and macrophages and their potential regulatory effects on MuSC transcriptional programs. Ligand activity was prioritized based on corrected AUPR scores using a curated MuSC gene set, and top-ranked ligands were visualized through ligand–target and ligand–receptor interaction heatmaps.

### Protein extract preparation from tissue homogenate

Muscle tissue was isolated using clean tools, kept on ice to prevent proteolysis, and processed as rapidly as possible. Tissues were snap-frozen by immersion in pre-chilled 2-methylbutane placed in liquid nitrogen for 1 min, then either stored at −80 °C or kept on ice for immediate processing. Approximately 5 mg of tissue was homogenized in 300 µl of complete extraction buffer consisting of 100 mM Tris (pH 7.4), 150 mM NaCl, 1 mM EGTA, 1 mM EDTA, 1% Triton X-100, and 0.5% sodium deoxycholate, with phosphatase and protease inhibitor cocktails and 1 mM PMSF added immediately before use according to the manufacturers’ instructions. Homogenization was performed using an electric homogenizer, with the blade rinsed twice with PBS and a third time with extraction buffer between samples. The resulting homogenates were agitated for 2 h at 4 °C on an orbital shaker, followed by centrifugation at 13,000 rpm for 20 min at 4 °C. The supernatant, containing the soluble protein extract, was aliquoted into chilled tubes, kept on ice during handling, and stored at −80 °C, with freeze-thaw cycles minimized to preserve sample integrity.

### Enzyme-linked immunosorbent assay (ELISA)

The protein extracts derived from tissue homogenate were used for enzyme-linked immunosorbent assay (ELISA) to quantify target proteins. Details of ELISA setup and reagents, including antibody dilutions and detection steps, were performed according to the manufacturer’s protocol (ELISA MAX™ Deluxe Set Mouse TNF-α, ELISA MAX™ Deluxe Set Mouse IL-1β, ELISA MAX™ Deluxe Set Mouse IL-6; BioLegend).

### MuSC isolation

Mice were euthanized, and tibialis anterior (TA) muscles dissected, minced, and digested in DMEM with Collagenase A (2 mg/ml), Dispase II (3 U/ml), DNase I (10 µg/ml) and 2% and BSA. Samples were incubated at 37 °C for 30 min with shaking. After centrifugation, the supernatant was filtered through a 40 µm cell strainer into FBS, rinsed with DMEM, and the cell suspension was re-incubated in fresh dissociation solution for another 30 minutes. Cells were then labeled with APC-anti-F4/80 (BioLegend; BM8), PE-anti-CD31(BioLegend; 390), PE-anti-CD45 (BioLegend; A20), FITC- anti-Ter119 (BioLegend; TER-119), PerCP-Cy5.5-anti-SCA1 (BioLegend; D7) for negative selection and BV421-anti-CD34 (BioLegend; SA376A4), APC-anti-ITGA7 (Thermo Fisher; 334908). Primers for qPCR were designed to amplify target genes including *Pax7* (Fwd: GACAAAGGGAACCGTCTGGAT; Rvs: TGTGAACGTGGTCCGACTG), *Myod1* (Fwd: CTCCAACTGCTCCGACGGCAT; Rvs: ACAGGCAGTCTAGGCTCGACAC), and the housekeeping gene *Tbp* (Fwd: CTACCGTGAATCTTGGCTGTAAAC; Rvs: AATCAACGCAGTTGTCCGTGGC).

### BMDM isolation

BMDMs were isolated as previously described.^48^^,^^49^ Isolated bone marrow-derived cells were differentiated for 6 d in the presence of L929 supernatant. Differentiated BMDMs were treated with IL-4 (20 ng/ml), LPS (100 ng/ml), Heme (20uM), INF-γ (20 ng/ml), INF-β(20 ug/ml) for 24 h. FACS labelling was performed using APC-anti-F4/80 (BioLegend; BM8) BV711-anti-MHCII (BioLegend; M5/114.15.2) and PE- anti-CD86 (BD bioscience; B7-2) 1:100 in PBS + 2%FBS.

### C2C12 culture in BMDM conditioned media

Bone marrow-derived macrophages (BMDMs) from control and Bach1-cKO mice were cultured in 150-mm dishes, with medium changes performed on days 3 and 5 of differentiation. On day 5, the medium was replaced with C2C12 differentiation medium (DMEM high glucose supplemented with 2% FBS, 1% insulin transferrin selenium as applicable, and 1% antibiotics). For inflammasome activation, BMDMs were primed with LPS (100 ng/ml) for 24 h, followed by ATP (5 mM) treatment for 15 min. After stimulation, the medium was immediately replaced with fresh C2C12 differentiation medium and collected as conditioned medium (CM) after an additional 24 h. After 24 h IL-1β neutralizing antibody (BioxCell; cat. no. BE0246) or isotype control (BioxCell; cat. no. BE0091)were added in 2 ng/µl or 5 ng/µl concentration. C2C12 myoblasts were maintained under proliferative conditions until approximately 70% confluence and then seeded at a density of 2,000 cells/cm^2^ onto collagen type I-coated 48-well plates. Cells were differentiated for 6 d using BMDM-derived conditioned medium, with medium replacement every 2 d. For immunofluorescence analysis, cells were fixed in 4% paraformaldehyde (PFA), permeabilized with 0.1% Triton X-100, and blocked with 2% BSA. Myogenic differentiation was assessed by staining with anti-Myosin Heavy Chain antibody (MF20; Invitrogen, cat. no. 53-6503-82) at 1:100 dilution overnight at 4 °C. When unconjugated MF20 was used, detection was performed with an Alexa Fluor 488-conjugated anti-mouse IgG secondary antibody. Nuclei were counterstained with DAPI or Hoechst 33342. Images were acquired using a Nikon Eclipse TS2R inverted fluorescence microscope equipped with a Photometrics CoolSNAP Dyno camera. Images. Were analyzed using Fiji/ImageJ software.

## Supplementary Material

vkag101_Supplementary_Data

## Data Availability

The single-cell RNA sequencing (scRNA-seq) datasets generated from non-chimeric control and Bach1-cKO mice are available under GEO accession number GSE294248, while the scRNA-seq data from the bone marrow transplant (BMT) experiments can be found under GSE294249. Additionally, the ATAC-seq data sets used in this study were re-analyzed from the publicly available dataset GSE129393, and the ChIP-seq data were obtained from SRR23683735.

## References

[1] Ortega‐Gómez A , Perretti M, Soehnlein O. Resolution of inflammation: an integrated view. EMBO Mol Med. 2013;5:661–674. 10.1002/emmm.201202382 23592557 PMC3662311

[2] Eming SA , Wynn TA, Martin P. Inflammation and metabolism in tissue repair and regeneration. Science (1979). 2017;356:1026–1030. 10.1126/science.aam7928 28596335

[3] Caballero‐Sánchez N , Alonso‐Alonso S, Nagy L. Regenerative inflammation: when immune cells help to re‐build tissues. Febs J. 2024;291:1597–1614. 10.1111/febs.16693 36440547 PMC10225019

[4] Martinez FO , Gordon S. The M1 and M2 paradigm of macrophage activation: time for reassessment. F1000Prime Rep. 2014;6:13. 10.12703/P6-13 24669294 PMC3944738

[5] Chazaud B. Inflammation and skeletal muscle regeneration: leave it to the macrophages!. Trends Immunol. 2020;41:481–492. 10.1016/j.it.2020.04.006 32362490

[6] Patsalos A , Tzerpos P, Wei X, Nagy L. Myeloid cell diversification during regenerative inflammation: lessons from skeletal muscle. Seminars Cell Dev Biol. 2021;119:89–100. 10.1016/j.semcdb.2021.05.005 PMC8530826 34016524

[7] Zhou Y et al Single-cell RNA landscape of intratumoral heterogeneity and immunosuppressive microenvironment in advanced osteosarcoma. Nat Commun. 2020;11:6322. 10.1038/s41467-020-20059-6 33303760 PMC7730477

[8] Yeo AT et al Single-cell RNA sequencing reveals evolution of immune landscape during glioblastoma progression. Nat Immunol. 2022;23:971–984. 10.1038/s41590-022-01215-0 35624211 PMC9174057

[9] Wang X , Zhou L. The many roles of macrophages in skeletal muscle injury and repair. Front Cell Dev Biol. 2022;10:952249. 10.3389/fcell.2022.952249 35898401 PMC9309511

[10] Wang X , Zhou L. The multifaceted role of macrophages in homeostatic and injured skeletal muscle. Front Immunol. 2023;14:1274816. 10.3389/fimmu.2023.1274816 37954602 PMC10634307

[11] Fabre T et al Identification of a broadly fibrogenic macrophage subset induced by type 3 inflammation. Sci Immunol. 2023;8:eadd8945. 10.1126/sciimmunol.add8945 37027478

[12] Patsalos A et al Spatiotemporal transcriptomic mapping of regenerative inflammation in skeletal muscle reveals a dynamic multilayered tissue architecture. J Clin Invest. 2024;134:e173858. 10.1172/JCI173858 39190487 PMC11473166

[13] Coulis G et al Single-cell and spatial transcriptomics identify a macrophage population associated with skeletal muscle fibrosis. Sci Adv. 2023;9:eadd9984. 10.1126/sciadv.add9984 37418531 PMC10328414

[14] Varga T et al Macrophage PPARγ, a lipid activated transcription factor controls the growth factor GDF3 and skeletal muscle regeneration. Immunity. 2016;45:1038–1051. 10.1016/j.immuni.2016.10.016 27836432 PMC5142832

[15] Patsalos A et al A growth factor–expressing macrophage subpopulation orchestrates regenerative inflammation via GDF-15. J Exp Med. 2022;219(1):e20210420. 10.1084/jem.20210420 PMC8635277 34846534

[16] Zhang X et al Bach1: function, regulation, and involvement in disease. Khanna KK, editor. Oxidative Med Cell Longevity. 2018;2018:1347969. 10.1155/2018/1347969 PMC6189649 30370001

[17] Tzerpos P et al BACH1 acts as a pioneer repressor to enable macrophage plasticity and adaptation across tissues and inflammatory contexts. Immunity 2026. In press. 10.1016/j.immuni.2026.06.022 42468527

[18] Jia M et al Deletion of BACH1 attenuates atherosclerosis by reducing endothelial inflammation. Circ Res. 2022;130:1038–1055. 10.1161/CIRCRESAHA.121.319540 35196865

[19] Hu Y et al BACH1 impairs hepatocyte regeneration after hepatectomy with repeated ischemia/reperfusion by reprogramming energy metabolism and exacerbating oxidative stress. Biochem Pharmacol. 2024;226:116377. 10.1016/j.bcp.2024.116377 38906228

[20] Yuan Z et al Knockdown of Bach1 protects periodontal bone regeneration from inflammatory damage. J Cell Mol Med. 2023;27:3465–3477. 10.1111/jcmm.17916 37602966 PMC10660620

[21] Di Domenico F et al Bach1 overexpression in Down syndrome correlates with the alteration of the HO-1/BVR-a system: insights for transition to Alzheimer’s disease. J Alzheimers Dis. 2015;44:1107–1120. 10.3233/JAD-141254 25391381 PMC4677575

[22] Wiel C et al BACH1 stabilization by antioxidants stimulates lung cancer metastasis. Cell. 2019;178:330–345.e22. 10.1016/j.cell.2019.06.005 31257027

[23] Liang Y et al Transcriptional network analysis identifies Bach1 as a master regulator of breast cancer bone metastasis. J Biol Chem. 2012;287:33533–33544. 10.1074/jbc.M112.392332 22875853 PMC3460454

[24] Davudian S , Mansoori B, Shajari N, Mohammadi A, Baradaran B. BACH1, the master regulator gene: a novel candidate target for cancer therapy. Gene. 2016;588:30–37. 10.1016/j.gene.2016.04.040 27108804

[25] Wang T et al Antioxidants stimulate BACH1-dependent tumor angiogenesis. J Clin Invest. 2023;133(20):e169671. 10.1172/jci169671 PMC10575724 37651203

[26] Padilla J , Lee J. A novel therapeutic target, BACH1, regulates cancer metabolism. Cells. 2021;10:634. 10.3390/cells10030634 33809182 PMC8001775

[27] Suzuki K et al Bach1 promotes muscle regeneration through repressing Smad-mediated inhibition of myoblast differentiation. Asakura A, editor. PLoS One. 2020;15:e0236781. 10.1371/journal.pone.0236781 32776961 PMC7416950

[28] Patsalos A et al The BACH1–HMOX1 regulatory axis is indispensable for proper macrophage subtype specification and skeletal muscle regeneration. J Immunol. 2019;203:1532–1547. 10.4049/jimmunol.1900553 31405954 PMC6736746

[29] Tidball JG. Regulation of muscle growth and regeneration by the immune system. Nat Rev Immunol. 2017;17:165–178. 10.1038/nri.2016.150 28163303 PMC5452982

[30] Forcina L , Cosentino M, Musarò A. Mechanisms regulating muscle regeneration: insights into the interrelated and time-dependent phases of tissue healing. Cells. 2020;9:1297. 10.3390/cells9051297 32456017 PMC7290814

[31] Wang Y , Lu J, Liu Y. Skeletal muscle regeneration in cardiotoxin-induced muscle injury models. IJMS. 2022;23:13380. 10.3390/ijms232113380 36362166 PMC9657523

[32] Minari ALA , Avila F, Oyama LM, Thomatieli-Santos RV. Skeletal muscles induce recruitment of Ly6C^+^ macrophage subtypes and release inflammatory cytokines 3 days after downhill exercise. Am J Physiol Regul Integr Comp Physiol. 2019;317:R597–R605. 10.1152/ajpregu.00163.2019 31411900

[33] Panduro M , Benoist C, Mathis D. T _reg_ cells limit IFN-γ production to control macrophage accrual and phenotype during skeletal muscle regeneration. Proc Natl Acad Sci USA. 2018;115(11):E2585–E2593. 10.1073/pnas.1800618115 PMC5856564 29476012

[34] Bergen V , Lange M, Peidli S, Wolf FA, Theis FJ. Generalizing RNA velocity to transient cell states through dynamical modeling. Nat Biotechnol. 2020;38:1408–1414. 10.1038/s41587-020-0591-3 32747759

[35] Nagy G , Bojcsuk D, Tzerpos P, Cseh T, Nagy L. Lineage-determining transcription factor-driven promoters regulate cell type-specific macrophage gene expression. Nucleic Acids Res. 2024;52:4234–4256. 10.1093/nar/gkae088 38348998 PMC11077085

[36] Wolf FA et al PAGA: graph abstraction reconciles clustering with trajectory inference through a topology preserving map of single cells. Genome Biol. 2019;20:59. 10.1186/s13059-019-1663-x 30890159 PMC6425583

[37] Cheng QJ et al NF-κB dynamics determine the stimulus specificity of epigenomic reprogramming in macrophages. Science (1979). 2021;372:1349–1353. 10.1126/science.abc0269 PMC8489855 34140389

[38] Morikawa M et al ChIP-seq reveals cell type-specific binding patterns of BMP-specific Smads and a novel binding motif. Nucleic Acids Res. 2011;39:8712–8727. 10.1093/nar/gkr572. 21764776 PMC3203580

[39] Patsalos A et al In situ macrophage phenotypic transition is affected by altered cellular composition prior to acute sterile muscle injury. J Physiol. 2017;595:5815–5842. 10.1113/JP274361. 28714082 PMC5577539

[40] Chaweewannakorn C et al Roles of IL-1α/β in regeneration of cardiotoxin-injured muscle and satellite cell function. Am J Physiol Regul Integr Comp Physiol. 2018;315:R90–R103. 10.1152/ajpregu.00310.2017 29513560

[41] Chen SE , Jin B, Li YP. TNF-alpha regulates myogenesis and muscle regeneration by activating p38 MAPK. Am J Physiol Cell Physiol. 2007;292:C1660–1671. 10.1152/ajpcell.00486.2006 17151142 PMC3099536

[42] Serrano AL , Baeza-Raja B, Perdiguero E, Jardí M, Muñoz-Cánoves P. Interleukin-6 is an essential regulator of satellite cell-mediated skeletal muscle hypertrophy. Cell Metabolism. 2008;7:33–44. 10.1016/j.cmet.2007.11.011 18177723

[43] Browaeys R , Saelens W, Saeys Y. NicheNet: modeling intercellular communication by linking ligands to target genes. Nat Methods. 2020;17:159–162. 10.1038/s41592-019-0667-5 31819264

[44] McKellar DW et al Large-scale integration of single-cell transcriptomic data captures transitional progenitor states in mouse skeletal muscle regeneration. Commun Biol. 2021;4:1280. 10.1038/s42003-021-02810-x 34773081 PMC8589952

[45] Perluigi M , Tramutola A, Pagnotta S, Barone E, Butterfield DA. The BACH1/Nrf2 Axis in brain in Down Syndrome and transition to Alzheimer disease-like neuropathology and dementia. Antioxidants. 2020;9:779. 10.3390/antiox9090779 32839417 PMC7554729

[46] Yan L et al Macrophage plasticity: signaling pathways, tissue repair, and regeneration. MedComm (2020). 2024;5:e658. 10.1002/mco2.658. 39092292 PMC11292402

[47] Yosef N , Regev A. Writ large: genomic dissection of the effect of cellular environment on immune response. Science (1979). 2016;354:64–68. 10.1126/science.aaf5453 PMC5111631 27846493

[48] Czimmerer Z et al The epigenetic state of IL-4-polarized macrophages enables inflammatory cistromic expansion and extended synergistic response to TLR ligands. Immunity. 2022;55:2006–2026.e6. 10.1016/j.immuni.2022.10.004 36323312 PMC9649892

[49] Daniel B et al The active enhancer network operated by liganded RXR supports angiogenic activity in macrophages. Genes Dev. 2014;28:1562–1577. 10.1101/gad.242685.114 25030696 PMC4102764

