# Supplementary figures and images for: BACH1 orchestrates macrophage state transitions to coordinate regenerative inflammation
Source: J Immunol. 2026 Jun 10;215(6):vkag101. doi: 10.1093/jimmun/vkag101 (PMC13253109; doi:10.1093/jimmun/vkag101)

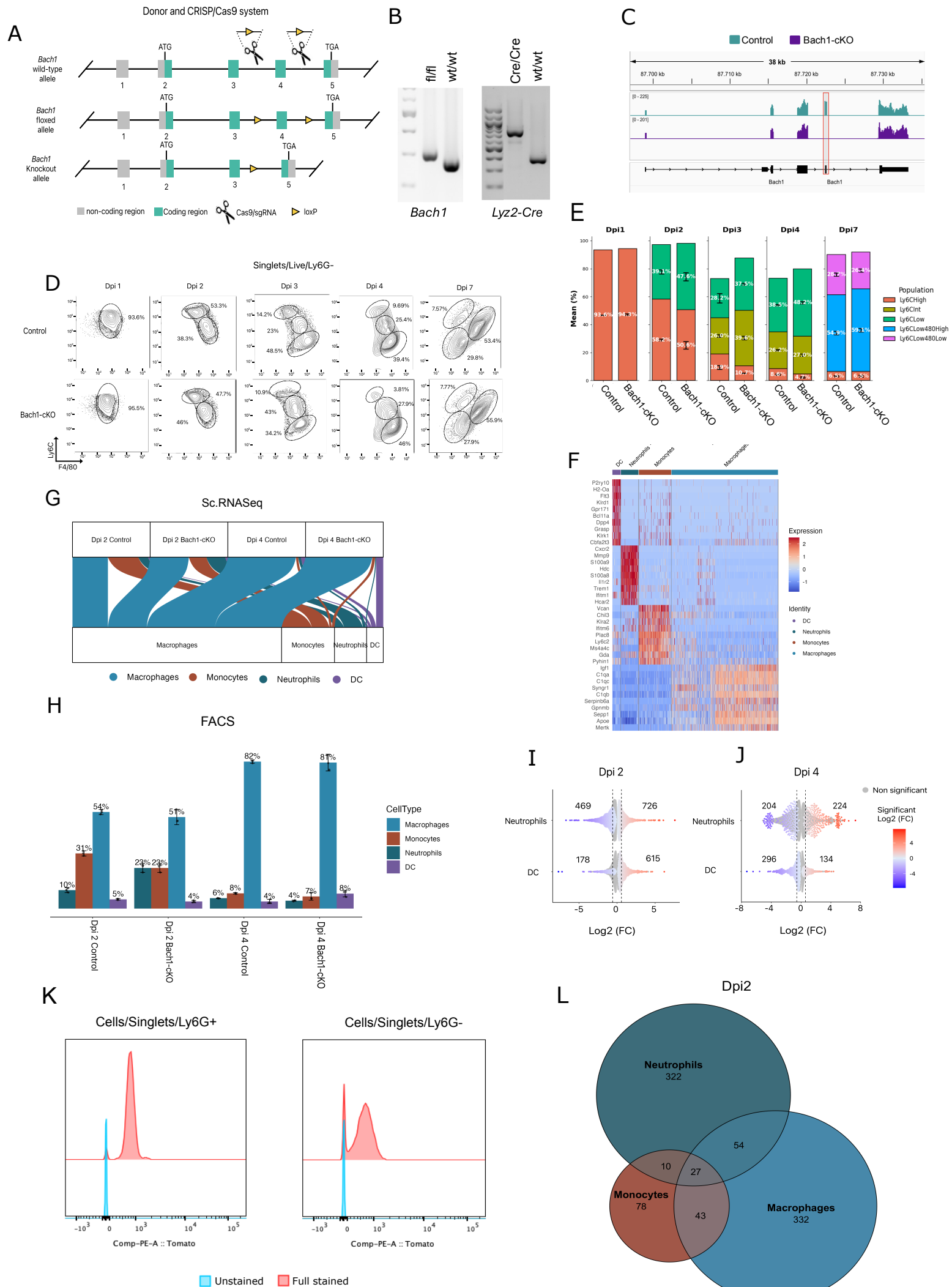

Supplement: vkag101_Supplementary_Data [file vkag101_supplementary_data.zip › 18-May-2026_014238_SupplmmentaryFigure1_Proof.pdf]

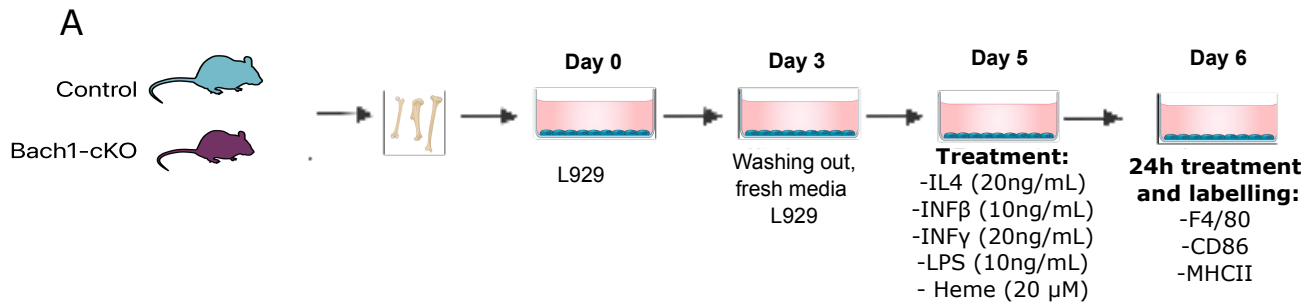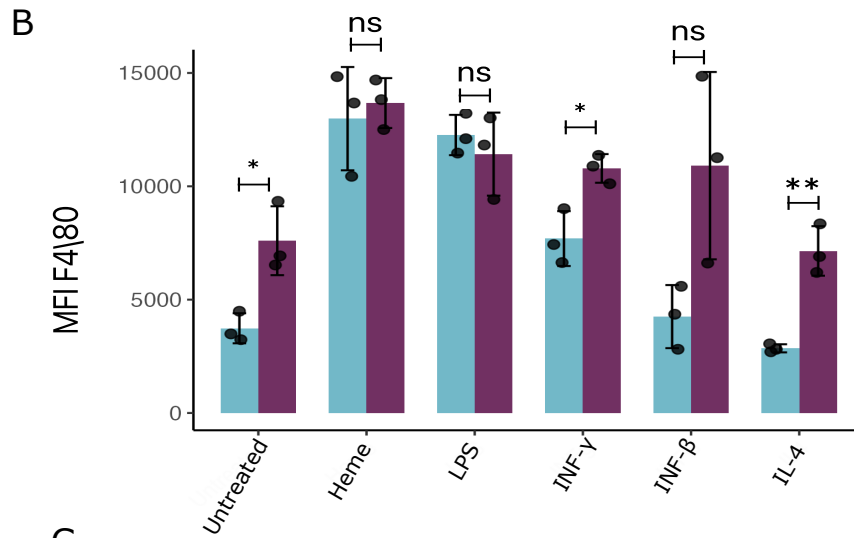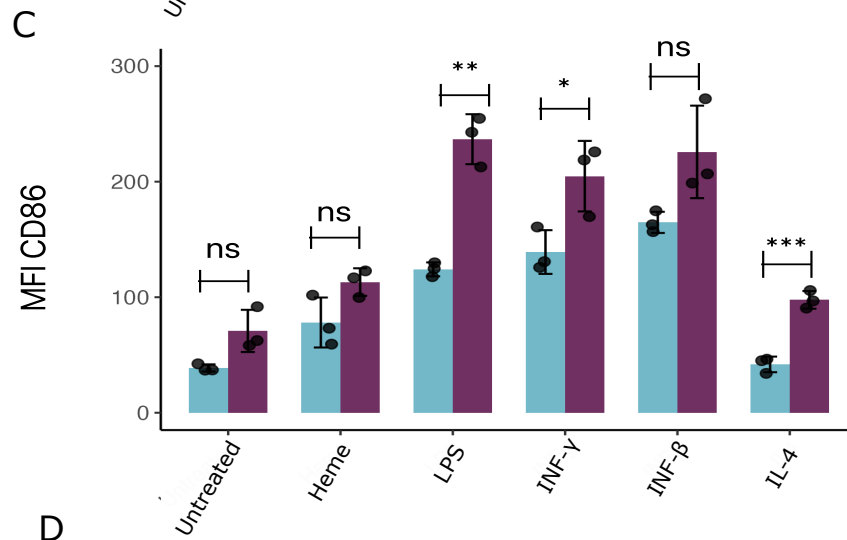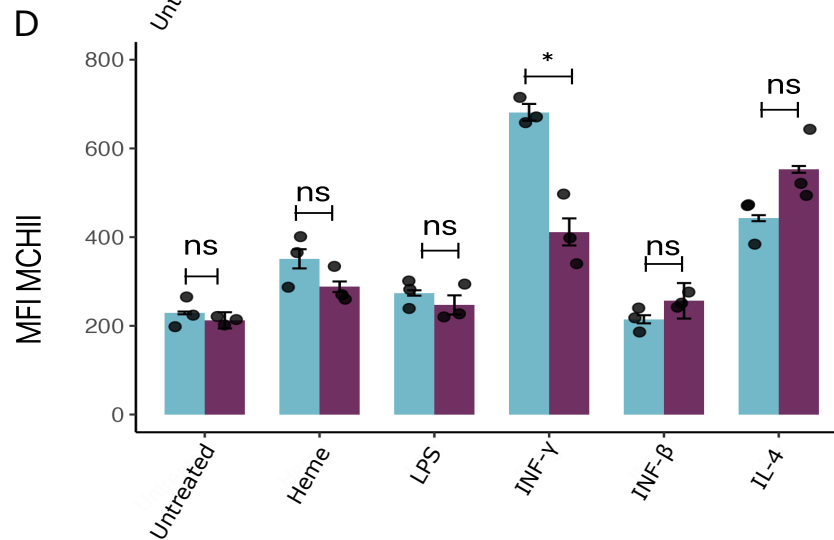

Supplement: vkag101_Supplementary_Data [file vkag101_supplementary_data.zip › 18-May-2026_014239_SupplementaryFigure4_Proof.pdf]
